# The diagnosis and management of extrauterine and uterine ectopic pregnancy

**DOI:** 10.1093/humupd/dmaf024

**Published:** 2025-10-08

**Authors:** Jessica Farren, Bassel H Al Wattar, Davor Jurkovic

**Affiliations:** Women’s Health Department, University College London Hospitals NHS Foundation Trust, London, UK; Elizabeth Garrett Anderson Institute for Women’s Health, Faculty of Population Health Sciences, University College London (UCL), London, UK; Clinical Trials Unit, Faculty of Health and Social Care, Anglia Ruskin University, Chelmsford, UK; Beginnings Assisted Conception Unit, Epsom and St Helier University Hospitals, London, UK; Women’s Health Department, University College London Hospitals NHS Foundation Trust, London, UK; Elizabeth Garrett Anderson Institute for Women’s Health, Faculty of Population Health Sciences, University College London (UCL), London, UK

**Keywords:** ectopic pregnancy, ultrasound, psychology, surgery, extrauterine ectopic pregnancy, interstitial ectopic pregnancy, ovarian ectopic pregnancy, abdominal ectopic pregnancy, uterine ectopic pregnancy, Caesarean scar ectopic pregnancy

## Abstract

In the last two decades, we have consolidated our knowledge of the epidemiology and risk factors for ectopic pregnancies. Minimally invasive surgical skills are now widespread, and laparoscopic surgery is recognized as the best and safest operative treatment for extrauterine ectopic pregnancies. Based on the evidence from randomized trials published a decade ago, laparoscopic salpingectomy is accepted as the optimal surgical treatment for tubal ectopic pregnancy. However, with recent advances in surgical techniques and improvement in surgical skills, the appropriateness of tubal removal versus conservation is under increasing scrutiny. Improvements in the organization and provision of care for women presenting with early pregnancy complications, in conjunction with better quality and wider use of ultrasound imaging, have resulted in an increased ability to detect small failing ectopic pregnancies, which were impossible to diagnose in the past. Many of these pregnancies are destined to resolve spontaneously without the need for any intervention. The necessity to avoid overtreatment and the potential for iatrogenic harm in such cases has facilitated the introduction of expectant management into mainstream clinical practice. This represents one of the key developments in the care for women with ectopic pregnancies. By contrast, the efficacy of medical management with methotrexate has been questioned. Another important development in recent years has been a rapid rise in the prevalence of ectopic pregnancies that are located outside the uterine cavity but within the confines of the uterus, the largest burden of which is from Caesarean scar ectopic pregnancies. This has promoted the development of new terminology and classification of ectopic pregnancies, with the aim of raising awareness of these increasingly prevalent types and minimizing the risk of misdiagnosis. In comparison to ectopic pregnancies outside the uterus, uterine ectopic pregnancies are more difficult to diagnose and manage, and are also associated with increased maternal morbidity, mortality, and adverse reproductive outcomes. Another challenge, which is peculiar to uterine ectopic pregnancies, is their potential to progress to reach foetal viability, albeit with a high risk of extreme prematurity. This requires women and clinicians to make difficult decisions about whether these pregnancies should be terminated to protect maternal health, despite some possibility of a good foetal outcome. Herein, we provide a comprehensive review of published literature to summarize new evidence and explore emerging themes with respect to ectopic pregnancy. Our aim is to provide an overview of modern classification and diagnosis, to summarize available treatment options and recommendations, and to emphasize longer-term outcomes, including the potential psychological impact of ectopic pregnancy. We examine current knowledge gaps and outline priorities for further research.

## Graphical Abstract

**Figure:**
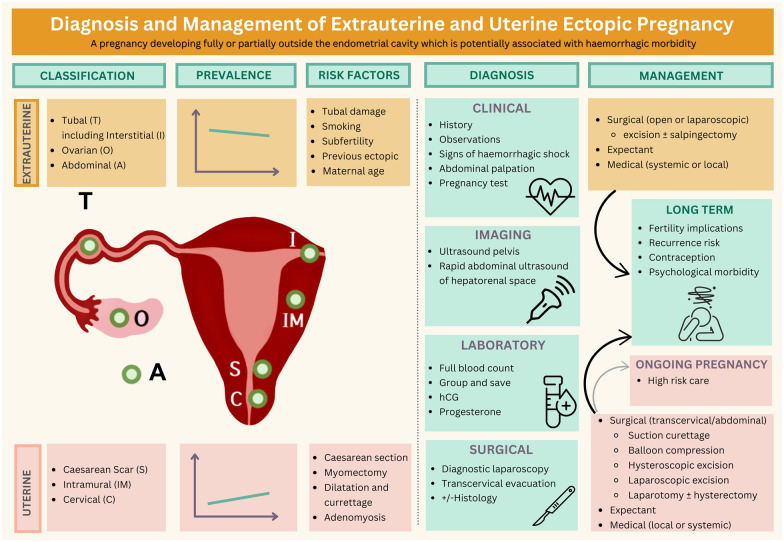
**The diagnosis and management of extrauterine and uterine ectopic pregnancy**.

## Introduction

An ectopic pregnancy can be defined as a pregnancy that develops fully or partially outside the endometrial cavity, and that, due to growth in an abnormal anatomical position, is potentially associated with haemorrhagic morbidity.

Bleeding and pain are common presentations in early pregnancy, with which women generally present sub-acutely, depending on access to early pregnancy services. While normal early pregnancy and miscarriage are the more common outcomes, ectopic pregnancy has scope for greater morbidity, especially if undiagnosed. The majority of ectopic pregnancies are located in the Fallopian tubes, and their diagnosis and management are a routine part of early pregnancy care. Ectopic pregnancies in other locations are less common, and it is thus more difficult for clinicians to acquire experience and skill to identify and treat them with confidence. Moreover, while most cases of ectopic pregnancy represent a clear deviation from normality, partial ectopic location across anatomical boundaries can make the differential diagnosis between normally sited and ectopic pregnancy very challenging (Kirk *et al.*, 2020). If a pregnancy is showing signs of growth and development, certainty in diagnosis is imperative to avoid inadvertent termination of a normal pregnancy.

In those with mild symptoms, or those in whom the diagnosis is uncertain, conservative management may enable resolution of this often self-limiting disease (RCOG, 2016; ACOG, 2018; NICE, 2023; Al Wattar *et al.*, 2024). In contrast, those with a certain diagnosis of progressive ectopic pregnancies will be recommended to have active intervention. Choice of management will depend on clinical symptoms and features of the pregnancy, such as its size, accessibility, and blood supply; competent assessment of these features is essential to ensure complexity is predicted, and referral to centres of expertize is made where needed.

Following resolution or treatment, it is important that the patient receives individual counselling about the risk of recurrence and appropriate support to plan future pregnancy. Clinicians should also be aware of the scope for psychological sequelae and the potential need for emotional support.

## Methods

Multiple literature searches to cover all aspects of this review were performed on Medline–PubMed to identify eligible English-language studies. The search period included all publications until July 2025, and search terms included ‘ectopic pregnancy’, ‘extrauterine pregnancy’, and ‘Ce(a)sarean scar pregnancy’. The reference lists of national guidances (American, European, and UK) and included articles were reviewed to identify additional qualifying studies. No formal quality assessments where made.

## Terminology and nomenclature

In 2020, an ESHRE working group of nine experts came together to reach consensus on good practice for terminology relating to ectopic pregnancy (Kirk *et al.*, 2020). An important first principle is that the term ‘ectopic’ should be used to refer to any pregnancy outside of the correct physiological location, as compared to ‘eutopic’ or ‘normally sited pregnancy’, which refers to a pregnancy sited within the boundaries set by the endometrial–myometrial junction. A recently published US multi-society panel recommended that the terms ‘intrauterine pregnancy’, ‘normally located pregnancy’, and ‘normally located intrauterine pregnancy’ could all be used to describe a pregnancy developing within the uterine cavity (Rodgers *et al.*, 2025) (Table 1). The problem with the term ‘intrauterine pregnancy’ is that it encompasses both normally sited pregnancies within the uterine cavity and uterine ectopics, which extend into the myometrium. In modern practice, where uterine pregnancies have become a frequent occurrence, the term ‘intrauterine pregnancy’ may be considered obsolete and could increase the risk of diagnostic errors.

**Table 1.** A comparison of the terminology proposed for the description of normal and ectopic pregnancy (EP) by (i) The European Society of Human Reproduction and Embryology (ESHRE) Working Group on Ectopic Pregnancy and (ii) The Society of Radiologists in Ultrasound Consensus Conference.

Definition	**ESHRE Working Group on Ectopic Pregnancy (Kirk *et al.*, 2020)**	**Society of Radiologists in Ultrasound Consensus Conference Recommendations (Rodgers *et al.*, 2025)**
Pregnancy implanted in a normal location in the uterine cavity, not extending beyond the endometrial–myometrial junction	Eutopic *or* Normally sited pregnancy *(The term ‘angular pregnancy’ should be abandoned)*	Intrauterine pregnancy *or* Normally located pregnancy *or* Normally located intrauterine pregnancy *or* Eccentrically located gestation sac completely surrounded by endometrium
Abnormal pregnancy location	Subtypes: Extra-uterine Tubal EP Interstitial EP Isthmic EP Ampullary EP Ovarian EP Abdominal EP Uterine EP Caesarean scar EP Cervical EP Intramural EP Within uterine anomaly	Subtypes: Tubal EP Interstitial EP Caesarean scar EP Cervical EP Ovarian EP Abdominal EP Intramural EP *(Terms related to morphological anomalies omitted)*
Tubal ectopic pregnancy with solid appearance, with variable echogenicity and vascularity	Solid swelling	Extraovarian mass *or* Adnexal mass
Tubal ectopic pregnancy containing a gestation sac	Containing a gestational sac ± yolk sac ± foetal pole	Tubal ring *or* Adnexal ring *or* Adnexal gestation sac
Ectopic pregnancy containing an embryo with visible heart pulsations	Live	With cardiac activity *(Discourage use of term ‘live’)*
Ectopic pregnancy showing ultrasound or biochemical signs of spontaneous resolution	Failing EP	*Not included*
Uterine or interstitial ectopic pregnancy in part within the uterine cavity	Partial	*No distinction between partial and complete uterine or interstitial EPs*
Uterine or interstitial ectopic pregnancy completely confined to myometrium with no visible connection to the endometrial cavity	Complete	
Ectopic pregnancy that remains visible on ultrasound after decline of serum hCG to pre-pregnancy levels	Residual ectopic pregnancy	*Not included*

An ectopic pregnancy is ‘live’ if there are visible heart pulsations and ‘failing’ if there is ultrasound or hormonal evidence of spontaneous regression. Again, the US recommendations are different (Table 1), and they discourage the use of the term ‘live’ in clinical practice (Rodgers *et al.*, 2025). This may have been driven by the desire to avoid the risk of this term being used to restrict women’s access to legal termination of pregnancy, which is a contentious political issue in the USA.

Extrauterine ectopic pregnancies can be tubal (including in the interstitial part of the tube), ovarian, or abdominal. Uterine ectopic pregnancies develop within the confines of the uterine visceral peritoneum and include Caesarean scar, cervical, and intramural pregnancies. They may also develop within a uterine anomaly.

A further important classification relates to whether all or part of the pregnancy breaches the anatomical boundaries. For example, where a uterine ectopic pregnancy has no visible connection to the uterine cavity, it is termed ‘complete’, but pregnancies that are part within and part outside the cavity should be termed ‘partial’.

Finally, a pregnancy can be ‘heterotopic’ when a correctly sited and an ectopic pregnancy co-exist. It is also possible to have two (or more) concomitant ectopic pregnancies.

Within this paper, we use the terms ‘woman’ and ‘women’ to refer to persons affected by ectopic pregnancy.

## Prevalence and mortality

Prevalence studies are hampered by a lack of national notification and surveillance infrastructures in both high-income and low- and middle-income countries (LMIC). Since many ectopic pregnancies resolve or progress without intervention, recorded prevalence reflects not only the actual trend of the condition, but also the availability of ultrasound and its sensitivity, which in turn reflects ever-improving technology and operator experience. It remains inevitable that many ectopic pregnancies are undiagnosed, and thus that the true prevalence of ectopics is higher than reported.

The Nurses’ Health Study II cohort (recruited in the USA in 1989 and followed up for 30 years) demonstrated that 1% out of 41 400 pregnancies were ectopic (Gaskins *et al.*, 2018). A UK study showed a 4.5-fold increase in the incidence of ectopics from 3.45 to 15.5 per 1000 maternities between 1966 and 1996. The upward trend continued until 1992, but the rates were stable in the last 4 years of the study (Rajkhowa *et al.*, 2000).

Data from the 2019 Global Burden of Disease study demonstrated a marginal reduction in age-standardized incidence rates of ectopic pregnancy between 1990 and 2019 overall, to 170/100 000 persons in 2019 (Zhang *et al.*, 2023). Peak incidence globally was in the 25–29-year age group, though with a shift towards a later peak (30–34 years) over time in high socio-demographic index areas.

Overall incidence of ectopic pregnancy following ART in the UK from 2000 to 2012 was 1.4%, with a significant decline in risk over this time (Santos-Ribeiro *et al.*, 2016). The risk of ectopic pregnancy associated with ART in the USA declined from 2 to 1.6% from 2001 to 2011 (Perkins *et al.*, 2015).

Tubal ectopic pregnancy (TEP) is the most common form of ectopic pregnancy and accounts for 95–97% of all cases (Bouyer *et al.*, 2002; Dooley *et al.*, 2019). The estimated prevalences of other types of ectopic pregnancies sit around 1–2% of ectopic pregnancies (excepting cervical and rudimentary horn pregnancies, which are rarer) (Bouyer *et al.*, 2002). However, the prevalence of uterine ectopic pregnancy can be expected to increase over time, reflecting both increased uterine surgery and increased recognition.

Historically, heterotopic pregnancies had been reported to occur in 0.003% (1:30 000) of spontaneous pregnancies and in 1% of pregnancies conceived via ART (Svare *et al.*, 1993; Tal *et al.*, 1996; Li *et al.*, 2013). More recent reports from the UK showed a prevalence of 0.05% (1:2000) in women attending early pregnancy units (Dooley *et al.*, 2020) and 0.04% (1:2500) in ART pregnancies (Santos-Ribeiro *et al.*, 2016), the latter probably in part reflecting the move towards single embryo transfers.

Thankfully, mortality from ectopic pregnancy is reducing overall, with the maternal mortality rate in the UK and Ireland from ectopic pregnancy reported at its lowest at 0.4 per 100 000 maternities in 2018–2020 (MBRRACE-UK, 2022). Global data have shown a similar pattern, with an age-standardized death rate in 2019 of 0.16/100 000 persons (Zhang *et al.*, 2023), though overall mortality remains significantly higher in LMIC. However, the MBRRACE report from 2021 to 2022 expedited a review of early pregnancy-related deaths due to a concerning increase in mortality to 0.8 per 100 000 maternities. Issues identified in the 12 deaths from ectopic pregnancy included delays in transfer to hospital and lack of face-to-face examination during the COVID-19 pandemic (MBRRACE-UK *et al.*, 2024b). There is also evidence of higher complications in women of racial and ethnic minority groups (Stulberg *et al.*, 2016).

## Risk factors, aetiology, and pathogenesis

### Risk factors for tubal ecoptic pregnancy

Established risk factors for TEP include antecedent tubal damage (due to infection, endometriosis, previous pelvic surgery, or smoking) and, as its corollary, a history of subfertility (Coste *et al.*, 1991; Saraiya *et al.*, 1998; Bouyer *et al.*, 2003; Li *et al.*, 2014; Davies *et al.*, 2016; Gaskins *et al.*, 2018; Farland *et al.*, 2019; Yong *et al.*, 2020). A positive chlamydia test was associated with a 30% increase in risk of ectopic pregnancy in a large cohort study from Denmark (Davies *et al.*, 2016); this association has been found to be significantly stronger in LMIC compared to high-income countries in one meta-analysis (Tang *et al.*, 2020). Gonorrhoea infection has also been strongly associated (Reekie *et al.*, 2019). TEPs are more common in older women (Raine-Bennett *et al.*, 2022), which could reflect the accumulation of other risk factors, anatomical changes, or increased incidence of chromosomal issues. There is also some evidence of inheritance: a Danish registry study demonstrated a 50% higher risk of TEP in daughters of women with an ectopic pregnancy (Karhus *et al.*, 2014).

The strongest association is with a past ectopic: after one TEP, studies have demonstrated a recurrence risk of between 5 and 19%, representing up to a 10-fold increase compared to the general population (Skjeldestad *et al.*, 1998; Banz *et al.*, 2010; Lund Karhus *et al.*, 2013; Mol *et al.*, 2014; Chouinard *et al.*, 2019). Prior miscarriage has been associated with an increased risk of ectopic pregnancy in some studies, but not in others (Nordenskjold and Ahlgren, 1991; Bouyer *et al.*, 2003; Gaskins *et al.*, 2018); since prior eutopic pregnancy implies tubal function and therefore lower risk, the possibility of shared aetiology should be considered.

Effective contraception will lower the risk of pregnancy, and therefore of TEP, overall. However, it is well recognized that if pregnancy occurs despite intrauterine contraceptive device (IUCD) use, it is significantly more likely to be ectopic (Gaskins *et al.*, 2018). Low-dose (13 mg levonorgestrel) hormonal IUCDs appear to be associated with a higher risk than all other forms of hormonal contraception (2.76 per 1000 women-years, compared to 0.3 for the 52 mg levonorgestrel IUCD or progestogen implant, 0.2 for combined oral contraception, and 0.24 for desogestrel 75 mg) (Kopp-Kallner *et al.*, 2022). Women who have used an IUCD in the past may be at higher risk of an ectopic pregnancy than never-users (Mol *et al.*, 1995; Bouyer *et al.*, 2003).

The use of emergency contraception (EC) has also been associated with TEP. Levonorgestrel EC is associated with adjusted odds ratios between 4.75 and 10.5 (Li *et al.*, 2015; Assouni Mindjah *et al.*, 2018; Shurie *et al.*, 2018). An association between ulipristal acetate EC and ectopic pregnancy has not yet been demonstrated (Levy *et al.*, 2014).

An association with Black ethnicity has been shown (Costa *et al.*, 1991; Raine-Bennett *et al.*, 2022), but the aetiological mechanisms and role of confounders remain unclear.

### Risk factors for other extrauterine ectopic pregnancy

Ovarian and abdominal pregnancies are also associated with tubal pathology (Yoder *et al.*, 2016; Chen *et al.*, 2023). IUCDs have been found to be strongly associated with ovarian ectopic pregnancies (OR 9.6 compared to non-users of contraception) (Sandvei and Ulstein, 1980; Zhu *et al.*, 2014). This may reflect the efficacy of the IUCD in preventing implantation diminishing with distance from it (Lehfeldt *et al.*, 1970). Chlamydia infection (OR 0.17), previous adnexal surgery (OR 0.25), and emergency contraceptive use (OR 0.24) have been associated with significantly lower odds of ovarian as compared to TEP (Zhu *et al.*, 2014).

### Risk factors for uterine ectopic pregnancy

The development of Caesarean scar ectopic pregnancy (CSEP) requires the presence of a myometrial defect, or niche. It has been shown that the risk of niche formation is higher in women with a retroflexed uterus and in those who have undergone multiple Caesarean sections (Ofili-Yebovi *et al.*, 2008). Surgical technique may also play a role, though the largest randomized study comparing single- and double-layer closure failed to demonstrate differences in the prevalence of niches between the two methods (Stegwee *et al.*, 2021).

Intramural pregnancy is caused by a breach, or abnormalities, of the endometrial–myometrial junction, which is often iatrogenic. They may result from previous myomectomy, classical Caesarean section, or trauma to the myometrium (usually perforation following uterine instrumentation) (Nijjar *et al.*, 2023). Intramural pregnancies have also been found within foci of adenomyosis (Ginsburg *et al.*, 1989).

In the past, the main risk factor for cervical ectopic pregnancy was a history of Caesarean section (Jurkovic *et al.*, 1996). Since CSEP has been recognized as a separate entity, the proportion of ectopics classified as cervical has dropped. In a recent study of 32 cervical pregnancies following IVF, a history of two previous pregnancies (OR 2.68), two or more miscarriages (OR 4.21), two prior dilation and curettage (OR 4.71), and smoking (OR 2.82) were identified as risk factors, but there was no association with previous Caesarean section or prior TEP (Matorras *et al.*, 2020). A strong association with dilation and curettage has also been shown in other studies (Hoyos *et al.*, 2019).

### Assisted reproduction and risk of ectopic pregnancy

IVF has been associated with all types of ectopic pregnancy (Marcus and Brinsden, 1993; Maymon *et al.*, 2004; Li *et al.*, 2014; Zhu *et al.*, 2014): indeed, the first IVF pregnancy in 1976 was tubal (Steptoe and Edwards, 1976). Risk increases with the number of embryos transferred, from 1.6% with a single embryo transfer, 1.7% for two embryos, and 2.5% when four or more are transferred (Perkins *et al.*, 2015).

Pre-existing factors, including tubal factor infertility and endometriosis, affect the risk of developing ectopic pregnancy following IVF. This is evidenced by lower risk in donor sperm-conceived pregnancies, in which tubal pathology is less likely (Allen *et al.*, 2021). The local pelvic environment at the time of embryo transfer also appears to play a role: the risk of ectopic pregnancy is lower following frozen–thawed embryo transfers, transfers without ovarian stimulation, and natural cycle IVF (Londra *et al.*, 2015; Zhang *et al.*, 2018; Jwa *et al.*, 2020). The risk is higher when the endometrium is thin (Rombauts *et al.*, 2015; Liu *et al.*, 2020; Zhao *et al.*, 2022).

Several studies link the risk of ectopic pregnancy to embryo status. Risk is higher with a Day 3 cleavage versus Day 5 blastocyst transfer (Zhang *et al.*, 2017; Zhang *et al.*, 2018; Trindade *et al.*, 2022), with lower-quality embryos (Anzhel *et al.*, 2022), and in women with reduced ovarian reserve (Lin *et al.*, 2017).

Ovulation induction has also been found to significantly increase the risk of ectopic pregnancy (Marchbanks *et al.*, 1985). An association between ovarian pregnancy and both ovulation induction and IUI has also been demonstrated (Fernandez *et al.*, 1991; Ko *et al.*, 2012).

### Aetiological mechanisms

Passage of the conceptus into the uterine cavity requires both anatomical patency of the tube and functional motility, which appears to rely predominantly on the cilia. A reduction in ciliary beat frequency has been demonstrated in laboratory studies of tubal epithelium exposed to high levels of progesterone (Mahmood *et al.*, 1998; Wanggren *et al.*, 2008; Bylander *et al.*, 2010; Zhao *et al.*, 2015). This may be mediated through downregulation of ‘transient receptor potential vanilloid 4’ channels (Li *et al.*, 2019). The proinflammatory cytokine interleukin 6 has also been found to reduce ciliary beat frequency, raising a mechanism by which endometriosis may amplify risk (Papathanasiou *et al.*, 2008). The impact of smoking may be mediated by prokineticin receptor 1, which in turn impacts smooth muscle contractility and receptivity markers such as leukaemia inhibitory factor (Shaw *et al.*, 2010).

Interleukins 6 and 8 have also been shown to increase the receptivity of the tubal epithelium to implantation, potentially via the expression of leukaemia inhibitory factor (Guney *et al.*, 2008). Adrenomedullin hormone has an anti-inflammatory effect, reducing the production of these interleukins, and its expression has been found to be reduced in patients with salpingitis (Wang *et al.*, 2020). Integrin B1, which may be associated with past chlamydia infection, also enhances tubal attachment (Ahmad *et al.*, 2018). Ovarian ectopic pregnancy has been associated with an inflammatory process within the ovarian stroma, with macrophages and mastocytes providing growth factors and activating angiogenesis (Istrate-Ofiţeru *et al.*, 2020).

There are wide variations in the proportion of TEPs reported to have chromosomal abnormalities, with some studies showing similar rates to eutopic pregnancies (Han and Yang, 1995; Goddijn *et al.*, 1996) and others showing higher levels of 24–33%, similar to miscarriage (Karikoski *et al.*, 1993; Toikkanen *et al.*, 1993). Studies have also demonstrated higher rates of aneuploidy in ectopic pregnancies with low hCG levels, in those without evidence of embryonic development, and in those that are unruptured (Elias *et al.*, 1981; Erel *et al.*, 1996; Block *et al.*, 1998), leaving open the question as to whether small TEPs that are conservatively managed (historically harder to diagnose, and less likely to require intervention necessary for karyotype analysis) may more commonly have genetic aetiology.

In IVF, the process of embryo transfer has been hypothesized to promote reverse migration. However, studies to adjust technical aspects of transfer in order to reduce risk have not been convincing (Halvaei *et al.*, 2013). One study demonstrated that tenaculum application stimulates uterine contraction and theorized that this might lead to retrograde movement of the embryo (Lesny *et al.*, 1999).

Caesarean scar, cervical, and intramural pregnancies tend to occur due to anatomical distortion, generally caused by previous surgery, which gives the conceptus access to an implantation site outside the confines of the endometrial cavity. Caesarean scar tissue shows evidence of inflammation and decreased smooth muscle volume density, and the hypoxic environment of scarred myometrium may stimulate preferential implantation and trophoblast invasion into the deep muscle layer (Dunwoodie, 2009; Roeder *et al.*, 2012; Lin *et al.*, 2023).

### Pathogenesis

As a TEP fails, it separates from its implantation site, similar to separation during miscarriage, which results in bleeding. The blood may form a haematosalpinx and may also be extruded into the peritoneal cavity, which irritates the peritoneal surface and causes pain. The bleeding is usually self-limiting, and overall blood loss is low or moderate.

In growing TEPs, a pro-angiogenic factor (WNT2B) produced by trophoblasts promotes villous vasculogenesis, angiogenesis, and vascular network expansion (Zhao *et al.* 2023). Catastrophic haemorrhage can ensue when these stretch and rupture their confines, due to thinning and necrosis of the tubal wall, or because of penetration of the trophoblast through the tube. Interstitial pregnancies may rupture later because of the greater thickness of the surrounding myometrium compared with the distal Fallopian tube and be associated with a higher risk of massive haemorrhage.

CSEPs implant in a region where there is absent decidua, with partial loss of the myometrium and the associated uterine vasculature. The pregnancy derives supply from larger, deeper, and higher-pressure vessels, resulting in an unstable pregnancy with high-velocity blood flow and placental lacunae (Jauniaux *et al.*, 2022). As a result, spontaneous subchorionic haemorrhage may result in loss of the pregnancy and massive bleeding. The fibrosed and deficient lower segment is unable to contract, further exacerbating blood loss. Despite being covered only by a very thin layer of tissue, rupture is a rare complication of CSEP, and live pregnancies have the potential to progress to full term.

Cervical pregnancies and intramural pregnancies are partially or completely implanted into the myometrium; in this respect, they are similar to CSEP. However, cervical pregnancies are located below the internal os, and their potential to develop beyond a very early stage is limited. The location of an intramural pregnancy, which can be anywhere within the myometrium of the uterine corpus, influences its clinical presentation. Their natural history differs from CSEP in that they often rupture despite being covered with a thick layer of vascular myometrium. This could be explained by the lack of superficial peritoneal scarring, which acts as a barrier to trophoblast invasion in CSEP.

## Clinical evaluation and diagnosis

Historically, the diagnosis of ectopic pregnancy was almost exclusively made post-mortem (Lurie, 1992). Key to earlier identification was the rapid immunological assessment of urine and serum to diagnose pregnancy, which became available in the 1960s (Marion and Meeks, 2012). Prior to the advent of accessible, high-quality ultrasound, laparoscopy was briefly considered the gold standard for diagnosis (Casikar *et al.*, 2012). Initially, an empty uterus on ultrasound scan (and high hCG) was a key indicator of ectopic pregnancy, but with ever-increasing resolution, positive identification of ectopic trophoblast is usually possible, with expert units describing between 74 and 83% of extrauterine EPs being positively identified on an initial scan (Kirk *et al.*, 2007; Dooley *et al.*, 2019). Ultrasound identification of ectopics remains a significant issue in low-income countries, where access to any healthcare, and specifically to ultrasound, may be limited, and presentation is often delayed until there are severe symptoms (Mooij *et al.*, 2018). Rarer subtypes, such as intramural and abdominal pregnancies, continue to pose a worldwide diagnostic challenge because most clinicians have scarce experience with them.

### Clinical assessment

The diagnosis of ectopic pregnancy must be considered in any woman of reproductive age attending a healthcare setting: failure to do so can have tragic consequences (MBRRACE-UK *et al.*, 2024b). The majority of women with ectopic pregnancy will present with mild or moderate clinical symptoms.

Evidence does not justify routine ultrasound assessment in asymptomatic women, though it may be considered for reassurance in those with a history of past ectopic pregnancy (Mol *et al.*, 2002). Pain or bleeding should prompt assessment: these symptoms are associated with a modest increase in the likelihood of TEP, with each additional day of bleeding beyond 3 days being associated with a 20% increase in risk (95% CI 14–27%) (Ayim *et al.*, 2016). However, attempts to produce a model to predict the likelihood of ectopic pregnancy based on pre-scan symptoms or risk factors have not been successful (Ayim *et al.*, 2016).

CSEP may be asymptomatic at presentation or may present with painless vaginal bleeding (Nijjar *et al.*, 2023). Similarly, cervical pregnancies are classically associated with painless vaginal bleeding. Ovarian ectopic may be more likely to present with pain alone (without vaginal bleeding), and with haemodynamic instability, with as many as 7% presenting with haemorrhagic shock (Zhu *et al.*, 2014; Melcer *et al.*, 2015; Zheng *et al.*, 2020; Li *et al.*, 2022; Solangon *et al.*, 2024).

In women of reproductive age, overt signs of haemodynamic compromise, including orthostatic hypotension, indicate that a significant circulating blood volume has already been lost, and further assessment must be escalated immediately. Abnormal vital signs without evidence of significant external blood loss are highly suggestive of a large intraperitoneal bleed. In LMIC, culdocentesis can be used to confirm the presence of intraperitoneal blood if there is no access to ultrasound (Hamura *et al.*, 2013). That aside, physical examination is of limited value in the prediction of ectopic pregnancy (Mol *et al.*, 1999). Certainly, speculum and digital vaginal examinations may be intrusive or painful, yet do not significantly add to the accuracy of the pre-scan diagnosis, so they cannot be recommended (Mol *et al.*, 1999). The latest enquiry into maternal deaths in the UK provided a reminder that clinical assessment of spontaneously expelled tissue is insufficient to confirm a correctly sited pregnancy (MBRRACE-UK, 2023). Decidua and decidual casts, along with clotted blood, are often impossible to distinguish macroscopically from trophoblast, and histological confirmation is always required.

The COVID-19 pandemic provided evidence of the importance of timely access to care; in one study, a higher proportion of women with TEP presented with haemoperitoneum (Dvash *et al.*, 2021), whilst in another, women presented with higher hCG levels, and first-line conservative management was more likely to fail (Kyriacou *et al.*, 2021).

### Imaging

Transvaginal ultrasound is an essential first-line investigation for ectopic pregnancy. In most cases, it can reveal the site of pregnancy and its morphological characteristics (Table 2). It can also detect any significant intraperitoneal bleeding. Transabdominal ultrasound may occasionally allow more accurate visualization, particularly if large fibroids obstruct transvaginal views or if significant adhesions cause axial orientation of the uterus. Importantly, all ultrasonography is operator dependent, and units must have established referral pathways for when there is diagnostic uncertainty.

**Table 2.** Ultrasound features of eutopic and ectopic pregnancies.

Diagnosis	Uterine shape	Interstitial tubes	Location	Communication between trophoblast and uterine cavity	Surrounding tissue	Mobility of pregnancy	Vascular supply	Schematic
**Eutopic pregnancies**								
**Eutopic**	Normal	2	Uterine cavity	Within uterine cavity	Continuous myometrial mantle	No	Surrounding endometrium	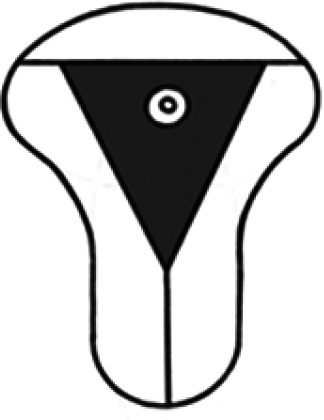
**Eutopic (within congenital uterine anomaly)**	Congenital uterine anomaly, e.g. septate, bicornuate, unicornuate	1 or 2	Uterine cavity	Within uterine cavity	Continuous myometrial mantle	No	Surrounding endometrium	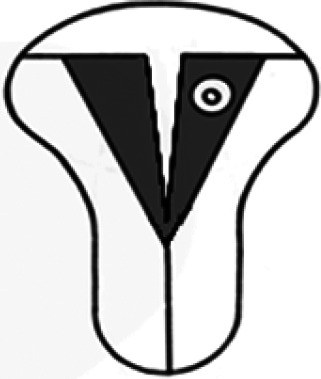
**Tubal ectopic pregnancies**								
**Tubal (isthmic, ampullary or fimbrial)**	Normal	2	Fallopian tube	Absent	Tube may be visible separately	Yes (historical adhesions or clot may limit)	Surrounding tube	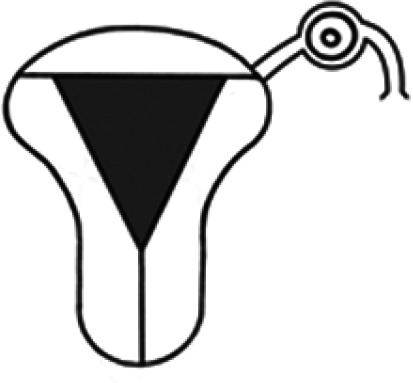
**Complete interstitial**	Normal	2	Interstitial portion of Fallopian tube	Narrow	Myometrial mantle, usually stretched	No	Surrounding myometrium	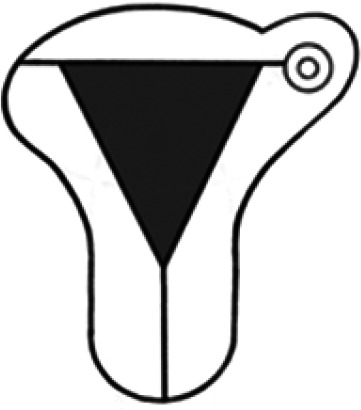
**Partial interstitial**				Wide	Myometrial mantle			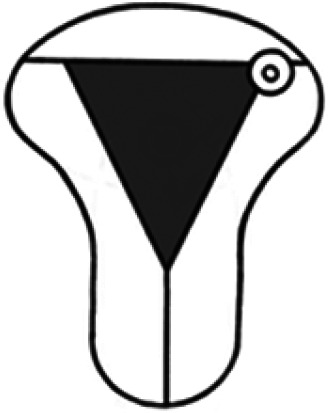
**Other extrauterine ectopic pregnancies**								
**Ovarian**	Normal	2	Ovarian stroma	Absent	Ovarian stroma	With ovary	Surrounding stroma	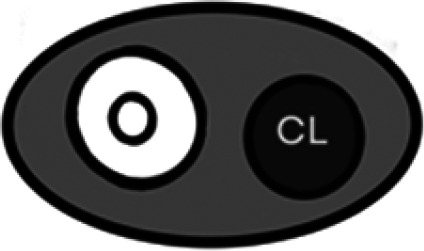
**Abdominal**	Normal	2	Anywhere in peritoneal cavity	Absent	Directly implanted onto peritoneal surface	No	Surrounding structures	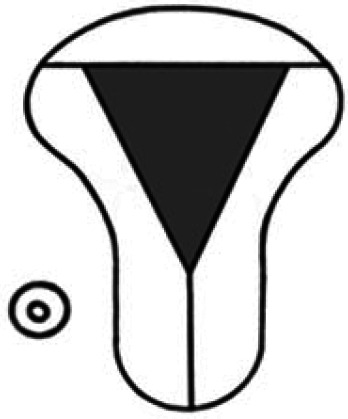
**Uterine ectopic pregnancies**								
**Partial Caesarean scar**	With Caesarean niche	2	Within niche	Wide	Myometrial defect, may herniate into broad ligament	No	Anteriorly within niche	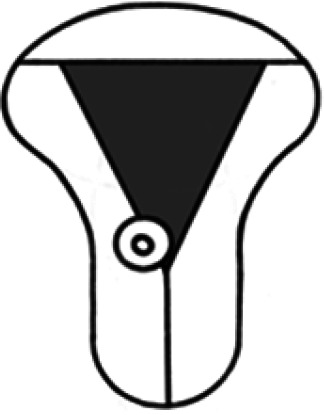
**Complete Caesarean scar**				Narrow or not visible				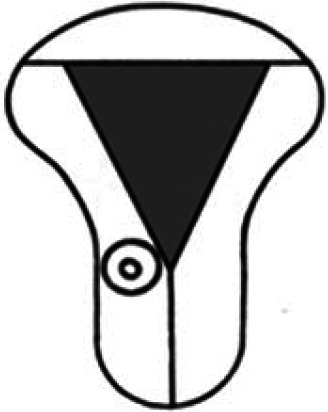
**Partial cervical**	Normal	2	Cervix; beneath uterine arteries	Wide	Cervical myometrium	No	Surrounding myometrium	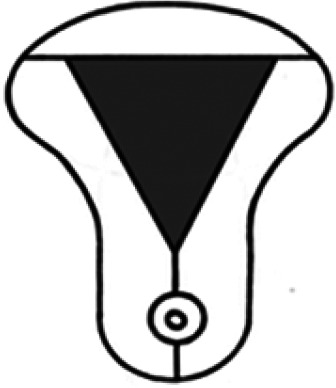
**Complete cervical**				Narrow or not visible				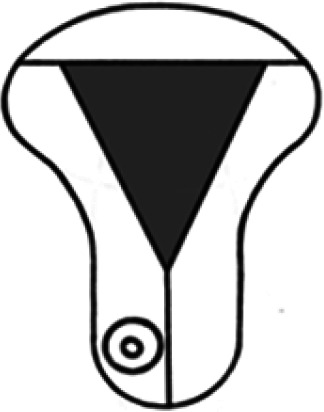
**Partial intramural**	Normal	2	Anywhere in myometrium—often displaces interstitial tubes	Wide	Myometrial mantle	No	Surrounding myometrium	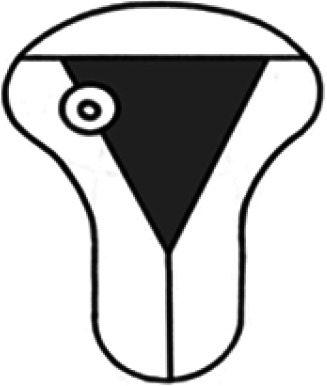
**Complete intramural**				Narrow or not visible				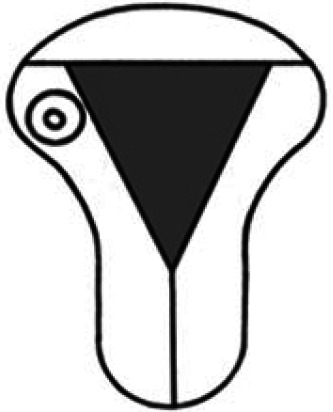
**Ectopic pregnancies within an anomalous uterus**								
**Non-communicating rudimentary horn**	Abnormal	1	Within rudimentary horn	Absent	Mantle of rudimentary horn, seen separate from the body of the unicornuate uterus	Yes (often marked)	Vascular pedicle between rudimentary horn and body of uterus	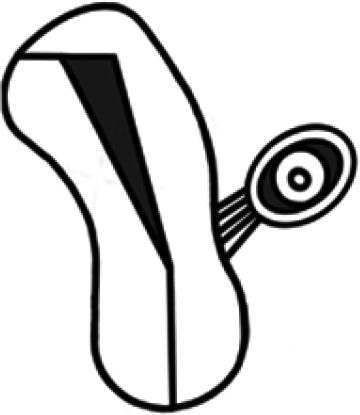
**Communicating rudimentary horn**		(second may be seen within rudimentary horn)	Within rudimentary horn	Variable	Mantle of rudimentary horn, fused with dominant horn	No	Surrounding endometrium	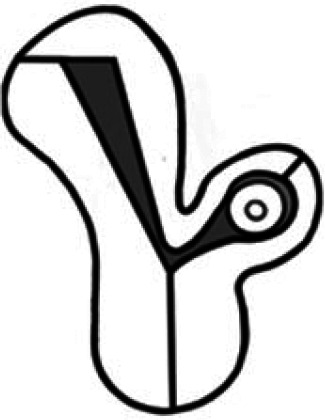
**Non-communicating hemicavity**	Abnormal	2	Within blind uterine horn	No communication with contralateral unicornuate cavity	Myometrial mantle	No	Surrounding endometrium	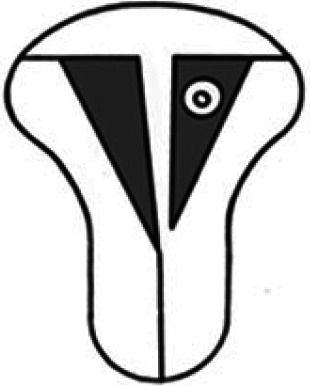

CL, corpus luteum.

We recommend a stepwise approach when performing ultrasound assessment, first assessing for uterine anomalies, and then assessing the communication between the trophoblast and uterine cavity. Ectopic pregnancies that occur in patients with a normal uterine shape (and two interstitial tubes) can also occur in those with uterine anomalies.

With improved quality of ultrasound equipment and better training, the ability to diagnose ectopic pregnancy has increased (Shalev *et al.*, 1998; Condous *et al.*, 2005; Dooley *et al.*, 2019). Unsurprisingly, detection rates improve with repeat scans: this reflects a higher index of suspicion and/or morphological progression over time (Farahani *et al.*, 2017; Nadim *et al.*, 2018; Dooley *et al.*, 2019).

It is important to recognize that achieving a true measure of the sensitivity of scanning is an unrealistic endeavour given the significant proportion that resolve without intervention (or, in the case of CSEP, develop into placenta accreta spectrum), and undoubtedly too without diagnosis. This means the ‘false negative’ rate is likely to be routinely underestimated.

Moreover, there is no agreed reference standard for ultrasound diagnosis of ectopic pregnancy. Appraisal of diagnostic performance generally involves comparing initial with later scans, or, for extrauterine pregnancies, looking at the subset that undergo surgical management. Although positive histology is sometimes viewed as the most reliable measure, consistently, 5% of those with established positive surgical findings have negative histology, with experts agreeing this reflects limitations of histological assessment rather than false-positive ultrasound examinations (Farahani *et al.*, 2017; Dooley *et al.*, 2019).

Using macroscopic surgical findings as the reference standard, false-positive diagnoses appear uncommon in surgically managed TEPs: 0.6% in the two largest studies, some of which may in fact have represented true TEPs that could not be confirmed surgically (Farahani *et al.*, 2017; Dooley *et al.*, 2019). There is no reliable measure of false-positive diagnoses amongst those expectantly or medically managed.

There is also no reliable measure of false-positive diagnoses of CSEP or interstitial pregnancy, which may result in inadvertent termination. As one indication of the magnitude of this issue, one published series retrospectively reviewed 20 eccentrically located pregnancies that had been diagnosed and treated as interstitial ectopics (Grant *et al.*, 2017). Ten (50%) were found to be completely surrounded by the endometrium and were re-classified as normally sited. This high potential for inadvertent termination of healthy normally sited pregnancies due to diagnostic errors should be recognized, and every effort should be made to prevent them.

#### Tubal ectopic pregnancy

**Figure 1.**
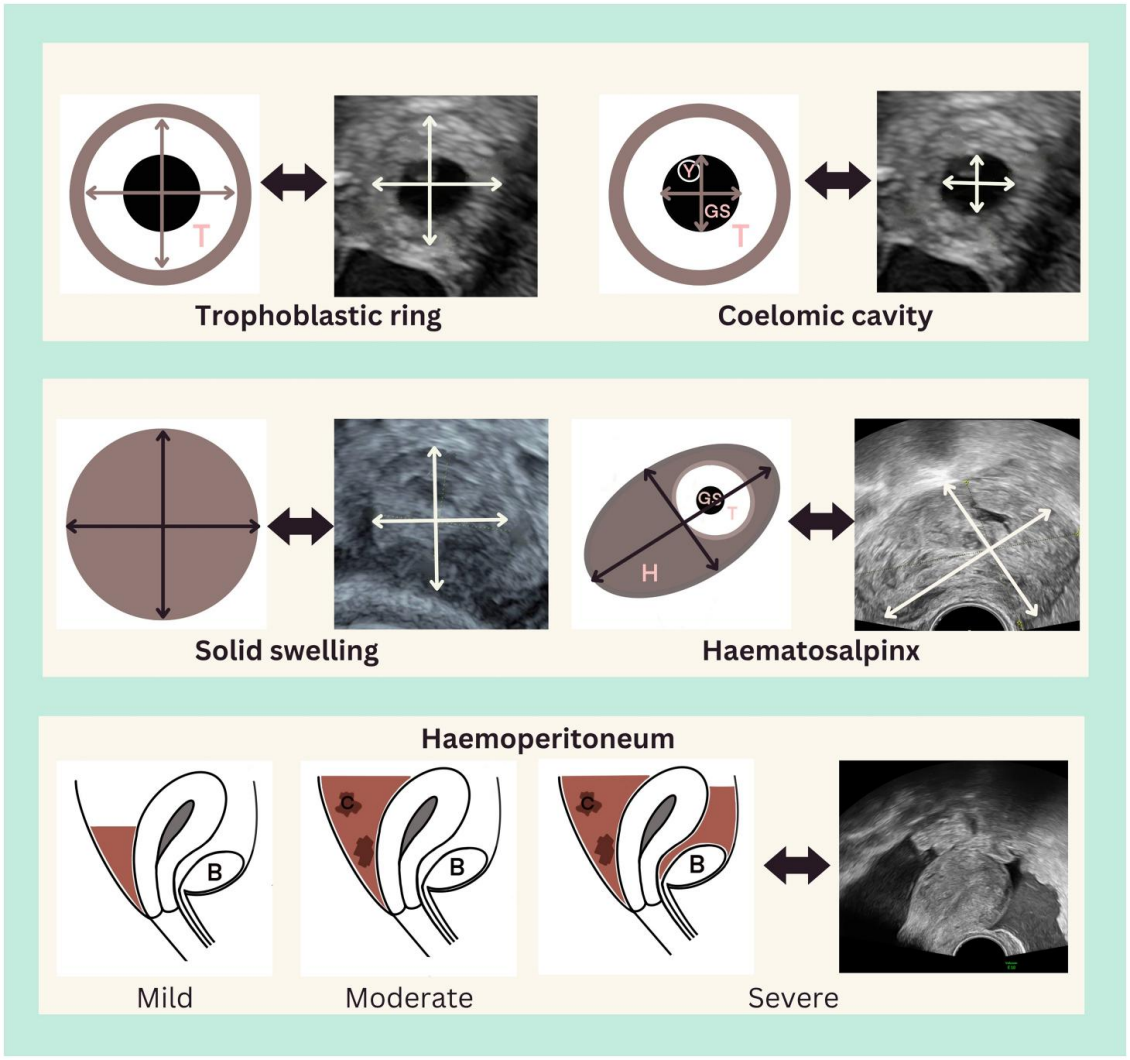
**Ultrasound measurement of tubal ectopic pregnancy and haematosalpinx, and assessment of haemoperitoneum**. All measurements are shown in two dimensions: a third orthogonal dimension should also be taken. Hyperechoic trophoblast (T) may be seen within the lumen of the tube, which may also contain a gestation sac (GS). There may be surrounding haematosalpinx (H). If structures, for example a yolk sac (Y) or embryo, are visible within the gestation sac, they should be measured in the same manner as in a eutopic pregnancy. Blood clot within the pelvis indicates moderate haemoperitoneum, and blood also in the uterovesical fold, next to the bladder (B), indicates severe haemoperitoneum.

The Fallopian tube has an interstitial portion (from the uterine tubal orifice to the outer edge of the myometrium), as well as an isthmic, ampullary, and fimbrial portion. Fimbrial ectopics can only be distinguished from ampullary ectopics during surgical treatment. TEPs are divided into different morphological subtypes, reflecting structures that can be seen on ultrasound: they can be inhomogeneous masses (also known as ‘solid swellings’ or ‘the blob sign’), or there can be clear structures within (a gestational sac ± yolk sac ± embryo). Morphology does not appear to have a significant impact on diagnostic accuracy (Nadim *et al.*, 2018; Dooley *et al.*, 2019). Common practice is to measure the overall size of the ectopic mass, encompassing the trophoblast, any haematosalpinx, and the tube itself. Such measurements will show the most correlation with surgical findings (Rajah *et al.*, 2018). However, where it is possible to differentiate the edges of the trophoblast ring, this should be measured, as should any structures visible within the ectopic (Fig. 1) (Rajah *et al.*, 2018).

Interstitial ectopics are completely or predominantly located within the intramural portion of the Fallopian tube. In the past, these pregnancies were referred to as ‘cornual’ pregnancies; this term has now been abandoned due to inconsistencies in its use (Kirk *et al.*, 2020). Although they may exist within the confines of the uterus, most interstitial pregnancies grow laterally into the proximal portion of the tube, justifying their inclusion within the subgroup of ‘extrauterine’ pregnancies, with which their management shares most similarity (Kirk *et al.*, 2020).

**Figure 2.**
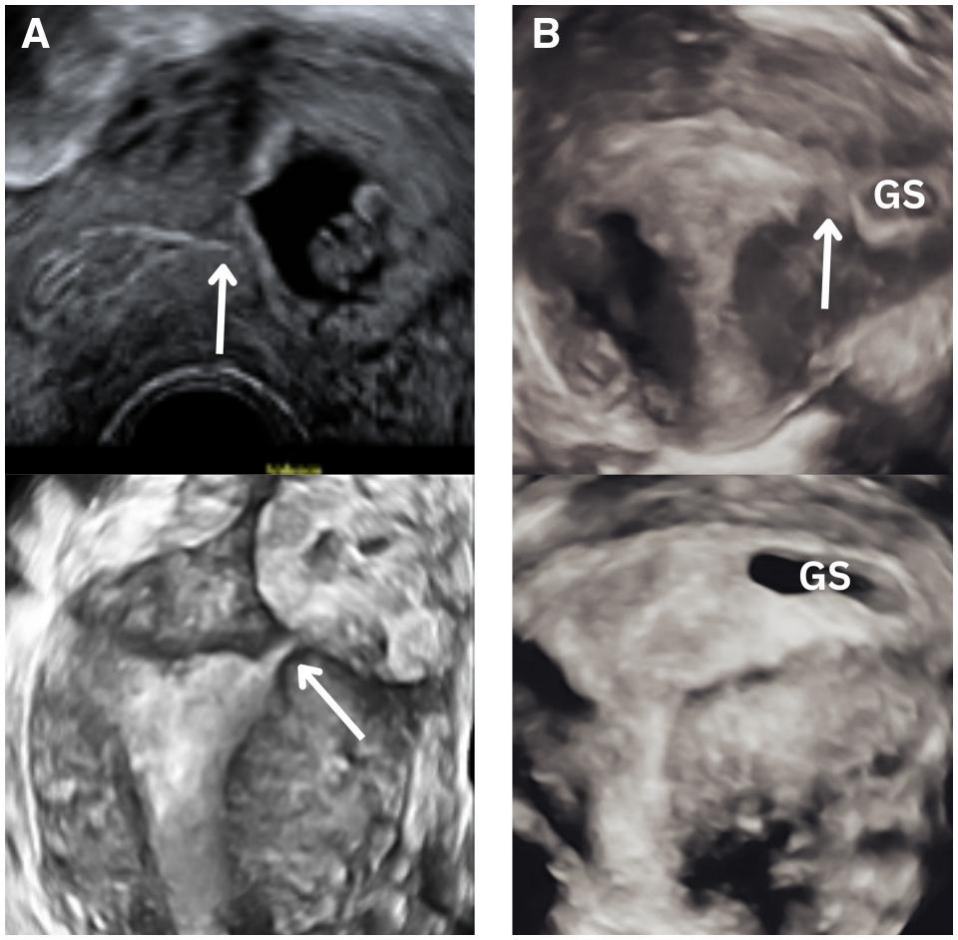
**Ultrasound images comparing complete interstitial ectopic pregnancy with a laterally located eutopic pregnancy**. (**A**) The gestational sac (GS) of a complete interstitial pregnancy, with the interstitial line demonstrated on both 2D and 3D ultrasound images by the white arrow. In (**B**), a laterally located eutopic pregnancy, 3D images demonstrate a laterally located eutopic pregnancy 1 week apart: in the top image the connection to the cavity (demonstrated with a white arrow) is not clear, leading to concern regarding potential interstitial location. However, as the GS expands, it is clear that it is eutopic. In cases of diagnostic uncertainty, repeating the scan at an interval is recommended to minimize the risk of false-positive diagnosis.

The pathognomonic feature of a complete interstitial pregnancy is the finding of an interstitial line between the gestation sac (GS) and the lateral aspect of the uterine cavity (Fig. 2) (Ackerman *et al.*, 1993). The myometrial mantle can be seen surrounding the sac but often appears thin laterally, and as gestation advances, a protrusion of the serosal surface of the uterus or extension into the isthmic part of the Fallopian tube will usually be visible sonographically and laparoscopically. The use of three-dimensional ultrasound to generate a coronal view of the endometrial cavity may be particularly helpful in demonstrating an eccentrically positioned gestation (Anandakumar and Mohammed, 2004). Interstitial pregnancies, and tubal pregnancies that sit in the proximal part of the tube, are commonly live, presumably reflecting the better vascular support at the site of implantation (Cassik *et al.*, 2005).

Interstitial pregnancies that grow medially and form a wider connection with the endometrial cavity represent ‘partial interstitial’ pregnancies; this diagnosis can be challenging to differentiate from a laterally implanted eutopic pregnancy, and thus any cases in which the interstitial line is not seen should be confirmed by an expert.

The corpus luteum is ipsilateral to a TEP in ∼7 out of every 10 cases; this may be helpful to take into account when searching for an ectopic pregnancy on an ultrasound scan (Ziel and Paulson, 2002).

#### Other extrauterine ectopic pregnancy

**Figure 3.**
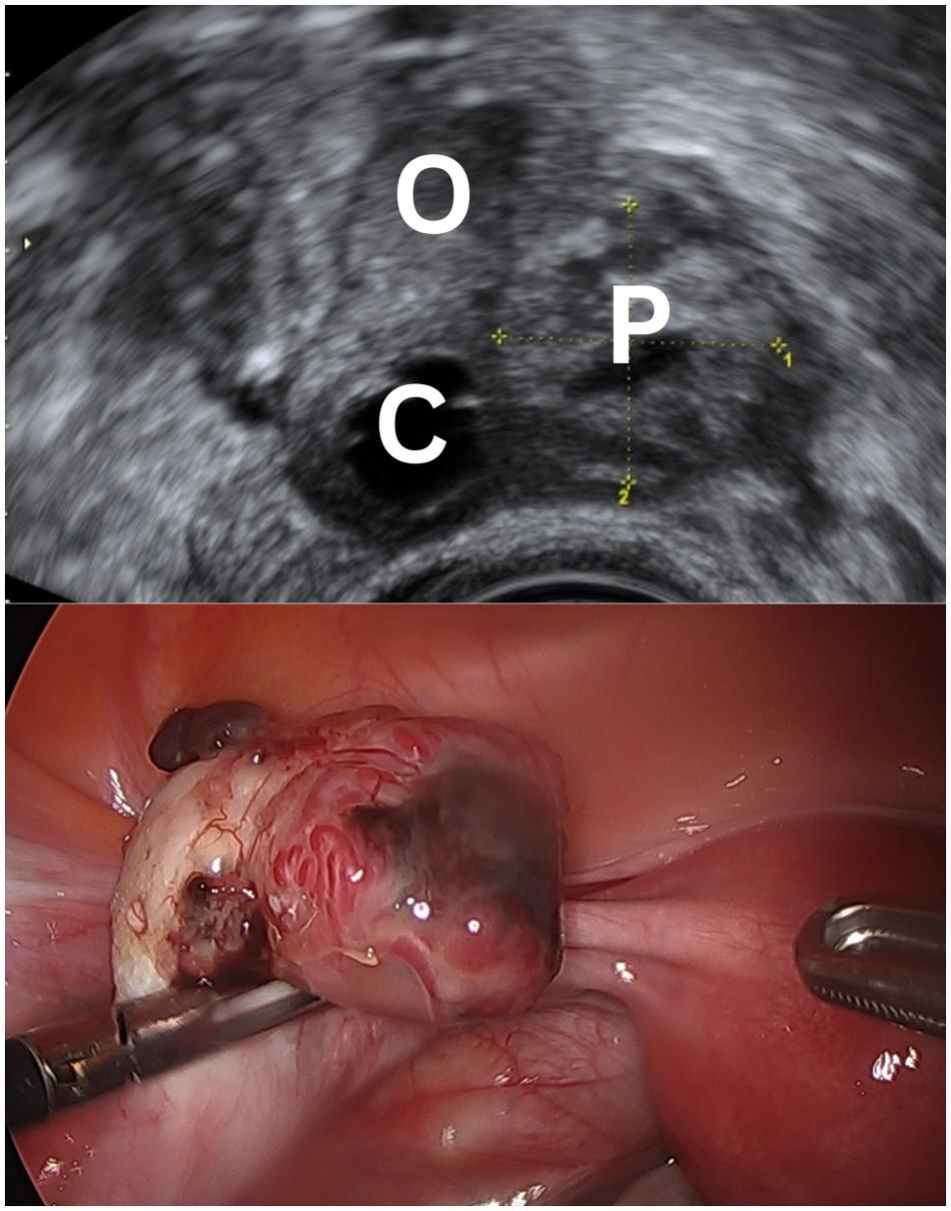
**Ultrasound and laparoscopic images of an ovarian ectopic pregnancy**. The ultrasound image demonstrates the ovary (O) containing a corpus luteum (C) and an ovarian ectopic pregnancy (P) with a defined hyperechoic trophoblast ring. At laparoscopy, the pregnancy can be seen protruding from the surface of the ovary. Resection of the ovarian ectopic was performed and the ovary was conserved.

An ovarian ectopic pregnancy can be seen on ultrasound as a gestational sac or a solid swelling within the ovarian parenchyma separate from the corpus luteum (Fig. 3). By comparison to the corpus, the ovarian ectopic will have a more defined, hyperechoic trophoblast ring, with less prominent (but still marked) blood supply, in contrast to the circumferential vascularity around a corpus. It has been reported that 80% of ovarian pregnancies are ipsilateral to the corpus luteum (Frates *et al.*, 2001; Comstock *et al.*, 2005; Solangon *et al.*, 2024).

Ovarian ectopic pregnancy is more likely to contain an embryo than TEP (Solangon *et al.*, 2024). The pregnancies, however, tend to be small for gestational age (Comstock *et al.*, 2005). The majority have haemoperitoneum at presentation (80% in one series of 79 patients) (Zheng *et al.*, 2020).

Misclassification of ovarian ectopic as TEP and vice versa appears common; in one study involving scans by expert practitioners, 5/20 ovarian ectopics were misdiagnosed as TEP and identified at surgery, and, over 10 years in which 1378 TEPs were diagnosed, there were four false-positive diagnoses of ovarian ectopic (Solangon *et al.*, 2024). All of these were TEP which were found to be adherent to the ovary at surgery.

Abdominal pregnancies are most commonly located on the serosal surfaces of the uterus and Fallopian tubes, or the broad ligament. However, pregnancies have also been reported involving the bladder, bowel, omentum, spleen, liver, and the retroperitoneal space (Poole *et al.*, 2012). Gerli *et al.* (2004) reported findings of a GS surrounded by loops of bowel, which may appear mobile on pressure with the transvaginal probe, as well as the absence of a correctly sited sac or a dilated Fallopian tube. Other authors have suggested, by contrast, that the GS will usually appear fixed, deep within the pelvis (Jurkovic and Mavrelos, 2007). Doppler examination will demonstrate surrounding vascularization. Beyond the first trimester, a failure to visualize the uterine wall between the foetus and the bladder, close proximity of the foetus to the abdominal wall, oligohydramnios, and the placenta located outside the uterine cavity are key ultrasound features (Allibone *et al.*, 1981). A pregnancy in one cornu of a bicornuate uterus could be misdiagnosed as an abdominal pregnancy (Angtuaco *et al.*, 1994). MRI may be helpful, especially where the implantation site is outside the pelvis, and in order to delineate the implantation of the placenta, particularly around the bowel or blood vessels (Lockhat *et al.*, 2006).

#### Uterine ectopic pregnancy

A CSEP is usually partial, with direct communication to the endometrial cavity. As it grows, it may be seen to predominantly progress towards the uterine cavity or towards the abdominal cavity. Transvaginal ultrasound is the primary imaging modality for the detection of CSEP, though concurrent transabdominal imaging with a full bladder may be helpful (Maymon *et al.*, 2004; Rotas *et al.*, 2006). MRI, though often used at later gestations for the related condition of placenta accreta spectrum, lacks the ability to assess dynamically for movement and blood flow and does not have a significant role in early pregnancy (Maymon *et al.*, 2004).

Ideally, the diagnosis of CSEP is made at 6–7 weeks’ gestation, before the GS expands into the endometrial cavity (Jordans *et al.*, 2022). A Caesarean scar defect, or niche, must be present (Jordans *et al.*, 2022).

A recent self-selected panel of experts defined the following criteria for diagnosis of CSEP (Verberkt *et al.*, 2024): (i) the uterine cavity and cervical canal are empty; (ii) the GS and/or placenta are in, or are in close contact with, the CS scar defect; (iii) the myometrial layer between the GS and the bladder wall or the anterior uterine wall is diminished; and (iv) there is evidence of abundant blood flow around the GS.

Multiple classification systems have been proposed (Lin *et al.*, 2018; Du *et al.*, 2020; Jordans *et al.*, 2022; Ban *et al.*, 2023). A group of experts reached consensus to classify the positioning of a GS in relation to the ‘uterine cavity line’ (located at the endometrial–myometrial junction) and the ‘serosal line’ (Jordans *et al.*, 2022). In a ‘Type 1’ CSEP, the largest part of GS crosses the uterine cavity line; in ‘Type 2’, the largest part is embedded in the myometrium but does not cross the serosal line; and in ‘Type 3’, the GS crosses the serosal line, sometimes with herniation into the vesico-uterine pouch or broad ligament. It is important to recognize that the pregnancy may change classification as it advances (Nijjar *et al.*, 2023). Furthermore, it should be considered for descriptive purposes only: the clinical relevance and prognostic value of this classification are unknown. The consensus group also advocated measurement of the ‘residual myometrial thickness’ in the sagittal plane, though again, the prognostic value of this is unknown. Placental lacunae are a common feature in 44% of CSEP, representing the effect of high-pressure vascular supply on the trophoblast (Jauniaux *et al.*, 2022).

Colour Doppler assessment allows delineation of the trophoblast and placenta from the scar and the bladder: this can help to localize implantation and should be considered an essential element of assessment (Nijjar *et al.*, 2023). High velocity (>20 cm/s) and low impedance (pulsatility index <1) waveforms are often seen (Jurkovic *et al.*, 2003). Doppler may also be essential to identify residual tissue after an incomplete miscarriage or the evacuation of a CSEP.

**Figure 4.**
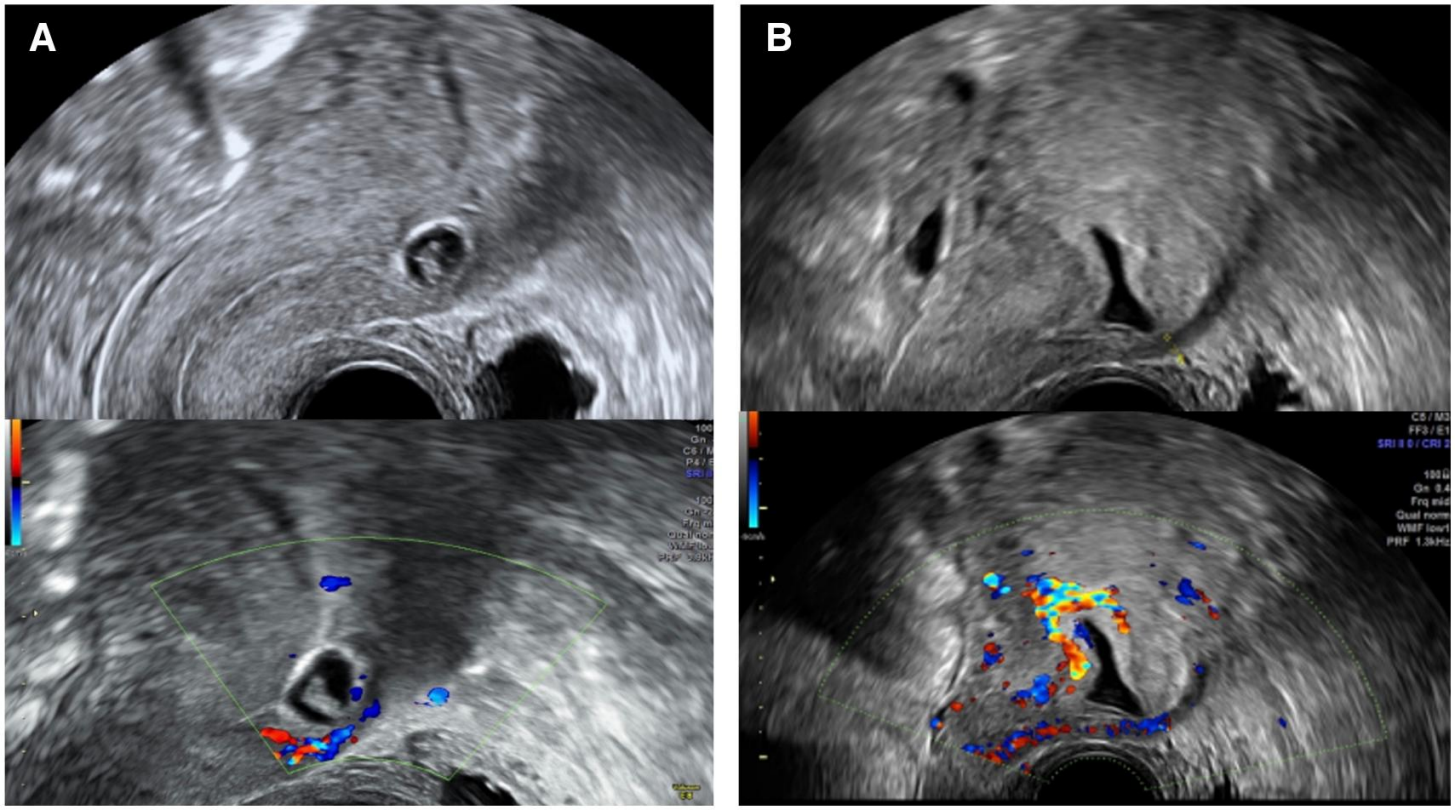
**Ultrasound images comparing true Caesarean scar ectopic pregnancy (CSEP) to eutopic pregnancy with posterior implantation**. These images originate from the same patient in two consecutive pregnancies after a Caesarean section. In (**A**), a true CSEP, the pregnancy is within the niche and blood supply is derived from the anterior uterine wall. In (**B**), a eutopic pregnancy with posterior implantation, Doppler examination reveals that the pregnancy is posteriorly implanted, and therefore does not represent a CSEP.

Importantly, pregnancies may be in close proximity to a Caesarean scar but not meet the diagnosis of a CSEP (Fig. 4). Low posteriorly implanted pregnancies may be seen to occupy space within the niche as they grow, but if they do not breach the endometrial–myometrial junction, they do not constitute CSEP (Kaelin Agten *et al.*, 2017).

The internal os (located at the level of the uterine artery) forms an important landmark for differentiating cervical (which lie below the internal os) from Caesarean scar (which usually lie at the internal os or above it) ectopic pregnancies. A cervical pregnancy will be considered partial if it communicates with the cervical canal and complete if it is surrounded completely by myometrium.

A potential source for misdiagnosis of both CSEP and cervical ectopic pregnancy is an active miscarriage: a GS may temporarily lodge near a Caesarean scar or in the cervix during expulsion. Dynamic assessment of movement of the sac within the cavity with gentle probe pressure (the ‘sliding sign’) and the absence of blood supply on Doppler assessment both suggest short-lived migration.

An intramural pregnancy will be seen to extend beyond the endometrial–myometrial junction, above the level of the internal os, and separate from the interstitial portion of the Fallopian tube (to which they are often closely located and may displace) (Kirk *et al.*, 2020; Nijjar *et al.*, 2023). Three-dimensional ultrasound has been shown to be an important adjunct to differentiate interstitial and intramural pregnancy (Nijjar *et al.*, 2023). Some intramural pregnancies will appear as heterogeneous solid lesions that may be difficult to differentiate from other uterine pathology, such as cystic fibroids or adenomyotic cysts. Doppler assessment of the blood flow is essential in these scenarios, with the trophoblast expected to generate significantly increased flow.

#### Ectopic pregnancy within a uterine anomaly

A unicornuate uterus is a congenital anomaly found in 1 in 500 women (Tellum *et al.*, 2023). There is usually a small, functional, non-communicating rudimentary horn (Nahum, 1998; Jayasinghe *et al.*, 2005). A pregnancy within this horn is associated with a high risk of rupture in the second trimester.

To diagnose a non-communicating rudimentary uterine horn ectopic pregnancy in a woman with a unicornuate uterus, the following criteria can be used (Jurkovic and Mavrelos, 2007): (i) a single interstitial portion in the main uterine body; (ii) a GS separate from the uterus, surrounded by myometrium; and (iii) a vascular pedicle joining the sac to the unicornuate uterus.

Communicating rudimentary horn ectopic pregnancies are very rare. Pregnancies within these may be considered ‘partial’, as they are in continuity with the endometrial cavity. This is likely to be one of the hardest diagnoses to confidently make within early pregnancy and can be complicated further as gestation advances and compensates for the asymmetry in size of the uterine cornua.

Other less common uterine anomalies also need to be considered, adding further complex locations for ectopic pregnancy. A Robert’s uterus is an asymmetric septate uterus where there is a blind hemicavity (Pfeifer *et al.*, 2021; Ludwin *et al.*, 2022). Similarly, a bicornuate uterus may have a blind hemicavity in which a pregnancy can implant.

#### Assessment of the endometrial cavity

Misclassifying an intrauterine fluid collection (often referred to as a ‘pseudosac’) or decidual cyst as a GS, and thereby missing an ectopic pregnancy, has scope to cause considerable harm. A pseudosac will follow the contour of the cavity and is surrounded by a single layer of tissue, in contrast to a GS, which will be surrounded by an echogenic ring and will be eccentrically positioned beneath an intact endometrial midline (Jurkovic and Mavrelos, 2007). Decidual cysts are usually at the junction of the myometrium and endometrium, are multiple, and do not have an echogenic rim.

Heterotopic pregnancies pose a particular risk; once a correctly located pregnancy is identified, examination of the adnexa may not be carried out with necessary diligence, and ectopic pregnancy may be missed. Systematic assessment for multiple corpora lutea and consideration of the number of embryos transferred during IVF aid in identifying those patients at risk.

Fibroids can distort the shape of the endometrial cavity, making confirmation of correct location much harder by preventing the GS and cervical canal from being visible in a single plane. Intrauterine adhesions may also complicate ultrasound appearances of an eutopic pregnancy, giving the appearance that a pregnancy is separated from the endometrial cavity.

Lateral implantation within the uterine cavity is a normal variant (Fig. 2) (Kirk *et al.*, 2020). The term ‘angular pregnancy’ should be abandoned, as it has potential for confusion with an interstitial pregnancy (Jansen and Elliott, 1981; Baltarowich, 2017; Kirk *et al.*, 2020). A case series of 42 such pregnancies confirmed that 80% resulted in a live birth, with no cases of uterine rupture or abnormal placentation (Bollig and Schust, 2020).

#### Assessment of haemoperitoneum

A proportion of patients with TEP will present with intraperitoneal blood. It is important to try and differentiate clinically and radiologically whether such fluid represents tubal rupture, with the potential for ongoing major haemorrhage, or spillage from the end of an intact Fallopian tube, associated with smaller and self-limiting quantities (Bignardi and Condous, 2009).

A useful semi-quantitative classification is proposed in the literature as follows: mild haemoperitoneum is considered any echogenic fluid in the pouch of Douglas; it is moderate if there are visible blood clots, and severe if clots and echogenic fluid are also present in the uterovesical fold (Fig. 1) (Rajah *et al.*, 2018). Clots represent a volume of blood that cannot be diluted sufficiently by peritoneal secretions to overcome clotting function.

It is also important to consider scanning the upper abdomen, especially in supine patients, with fluid in the hepatorenal space (Morrison’s pouch) indicating severe haemoperitoneum (>670 ml in a supine patient) (Abrams *et al.*, 1999). Such views of the right upper quadrant taken at the point of care in emergency departments is increasingly recognized as a useful tool for triaging unwell patients, and expediting surgical management (Moore *et al.*, 2007; Stone *et al.*, 2021).

### Biochemical assessment

It was initially believed that an ectopic pregnancy could be diagnosed with accuracy if a single hCG reading was above a certain level and a correctly sited pregnancy could not be seen. This concept of a ‘discriminatory zone’ was based on the assumption that a normal, correctly sited pregnancy should be seen on a transvaginal ultrasound scan with hCG >1500 IU/l or a transabdominal scan with hCG >6500 IU/l (Kadar *et al.*, 1981; Romero *et al.*, 1988; Cacciatore *et al.*, 1990). However, later studies showed that this concept was fundamentally flawed. It has been well documented that an ectopic pregnancy can be diagnosed and can cause morbidity at any hCG level, with a majority presenting with hCG <1500 IU/l (Counselman *et al.*, 1998; Mavrelos *et al.*, 2013). Moreover, false-positive diagnoses would be common following miscarriage (when hCG levels stay high for many days after a pregnancy has been expelled), in early multiple pregnancies, and pregnancies in large fibroid uteri (in which a small GS may be hard to visualize). There is, in fact, no safe hCG cut-off, which excludes a potentially viable eutopic pregnancy (Bobdiwala *et al.*, 2022).

Serial hCG measurements are considered to be more useful and are widely used. Although an abnormally slow rise in hCG is common in ectopic pregnancy, similar trends can also be seen in cases of early miscarriage. It has also been shown that in 10–20% ectopic pregnancies, serum hCG changes mimic normal eutopic pregnancies (Fridstrom *et al.*, 1995; Silva *et al.*, 2006). A meta-analysis of published studies concluded that serial hCG measurements cannot reliably discriminate between normally and abnormally located pregnancies (van Mello *et al.*, 2012).

Serum progesterone is produced by the corpus luteum; its level is determined by the changes in hCG levels (Duncan, 2021). Its half-life is short, and therefore its concentration in blood changes relatively rapidly in response to variations in hCG production. In clinical practice, single serum progesterone has been used as an alternative to serial hCG to predict whether the pregnancy is likely to progress or whether it is already in regression, but importantly there is no safe cut-off to exclude a potentially viable eutopic pregnancy (Verhaegen *et al.*, 2012; Bobdiwala *et al.*, 2022).

For these reasons, it is inappropriate to use serum hCG or progesterone alone to diagnose ectopic pregnancy or as a threshold for intervention without prior localization on ultrasound (Condous *et al.*, 2005). There is a risk of significant iatrogenic harm if either of these practices is adopted, and treatment should not be commenced in a clinically stable patient until the location of the pregnancy has been confirmed (Jin *et al.*, 2024).

Appropriate use of biochemical assessment occurs only after a competent ultrasonographic assessment has taken place, to rationalize follow-up. If a pregnancy is not visualized, it is referred to as a ‘pregnancy of unknown location’ or ‘PUL’. The proportion of PULs in individual units is determined by the configuration and quality of the ultrasound service and can vary between 7% and 47% (Memtsa *et al.*, 2020). The proportion of ectopic pregnancies in those initially presenting with PUL is also variable, and it tends to decrease with increasing rate of PUL.

Evidence surrounding the interpretation of hCG and progesterone levels for PUL is summarized in Table 3. Two important aspects of any follow-up protocol are: (i) reducing unnecessary follow-up in those whose history and biochemical assessment are suggestive of resolving failed pregnancies and (ii) prompting early re-assessment in those whose tests indicate significant risk of an undetected ectopic pregnancy with the potential to cause harm.

**Table 3.** Interpretation of hCG and progesterone levels following a scan in which a eutopic or ectopic pregnancy cannot be visualized (a pregnancy of unknown location).

Scenario	Hormone level	Interpretation	Reference
Pregnancy of unknown location	Progesterone ≤2 mmol/l	<2% diagnosed with EP: study proposes UPT in 2 weeks	Bobdiwala *et al.* (2020)
	Progesterone ≤10 nmol/l	∼2% risk of intervention: study proposes discharge after single visit	Cordina *et al.* (2011)
	Progesterone ≤20 nmol/l	∼4% risk of intervention: study proposes serum hCG or UPT in 1 week	Day *et al.* (2009)
Pregnancy of unknown location after IVF	hCG ≤100 IU/l at ≥5 weeks’ gestation hCG/C ratio <4 (progesterone levels cannot be interpreted in the context of progesterone supplementation)	2% risk of intervention: study proposes discharge after single visit	Dooley *et al.* (2024)

C, days from conception date (date of oocyte retrieval in fresh cycles) to date of presentation; EP, ectopic pregnancy; UPT, urinary pregnancy test.

The progesterone level is helpful to identify patients with failing pregnancies who are at low risk of complications and who are unlikely to require any form of medical intervention (Day *et al.*, 2009). Approximately one-third of patients with PUL will have progesterone <10 nmol/l, and a tiny proportion of these (4/227, 1.7% in one study) will need further treatment; by discharging these patients after their initial visit, early pregnancy services can run more effectively and social and professional disruption to the patients can be minimized (Cordina *et al.*, 2011). However, exogenous progesterone administration as part of IVF regimens or for miscarriage prevention is common, and different diagnostic algorithms should be used in this population (Dooley *et al.*, 2024). For patients with progesterone levels above the chosen low-risk cut-off on initial tests, repeat testing of hCG will indicate the rate of growth or regression of the pregnancy and rationalize repeat imaging or follow-up. Logistic regression models relying on the rate of change in hCG over 2 days have been developed and refined in order to standardize follow-up (Bobdiwala *et al.*, 2020). A combination of a high hCG and progesterone level may indicate a missed ectopic pregnancy with potential for harm, and the history and imaging should be reviewed by an experienced clinician, with a view to repeat the scan.

Live ectopic pregnancies identified on ultrasound generally do not require biochemical assessment: the hCG and progesterone levels will invariably be high, and surgical management is indicated.

#### Novel biomarkers

Potential alternative biomarkers include markers of trophoblast growth (such as human placental lactogen and inhibin A), of smooth muscle damage (such as smooth muscle heavy chain myosin and creatinine kinase), and of inflammation (such as interleukins 6 and 8). There has been extensive research on a multitude of such markers (Rausch and Barnhart, 2012; Daponte *et al.*, 2013). However, in prospective clinical studies, none have demonstrated sufficiently accurate prediction of diagnosis or resolution when used alone (Rausch and Barnhart, 2012; Memtsa *et al.*, 2020). Markers of trophoblast growth struggle to differentiate between miscarriage and ectopic pregnancy, and markers of smooth muscle damage or inflammation may only confirm that a pregnancy is at imminent risk of rupture. There is scope for future utility in establishing diagnosis or guiding management by combining multiple biomarkers or combining biomarkers and ultrasound markers; although case–control studies show promise, prospective studies are needed (Rausch *et al.*, 2011; Senapati *et al.*, 2016).

### Surgical assessment

In modern practice, surgery is rarely used as a primary diagnostic tool for ectopic pregnancy, but may be used in clinically unstable patients, or, occasionally, in cases where the diagnosis is not confirmed on ultrasound but the suspicion based on serum biochemistry is high. At laparoscopy, most TEPs present as swellings of the Fallopian tube, which can vary in size from a few millimetres to several centimetres. If the TEP has ruptured, the pregnancy tissue may be covered by blood clots and hard to find, but the site of rupture is usually identifiable.

However, even when used as a secondary test, there have been reports of both false-negative and false-positive findings at laparoscopy (Farahani *et al.*, 2017; Dooley *et al.*, 2019).

False-negative diagnoses are more likely to occur in the presence of severe pelvic adhesions. It is also often difficult to visualize small ectopics in the isthmic and interstitial tubes. Tubal abnormalities such as hydrosalpinx are a cause of false-positive diagnoses.

Uterine ectopic pregnancies may be missed during ultrasound assessment and only suspected at surgical management of a miscarriage or surgical termination; excessive blood loss may indicate that the pregnancy was implanted in the cervix or a Caesarean scar. Sometimes, the first consideration of intramural pregnancy occurs at attempted evacuation of a presumed eutopic pregnancy when tissue is not obtained (Nijjar *et al.*, 2023).

### Histological assessment

Surgically excised ectopic pregnancies are usually histologically examined, subject to consent from the patient, both to confirm the diagnosis and to assess for molar gestation. Trophoblast may sometimes be difficult to find after rupture or spontaneous extrusion of a pregnancy from the end of a tube, leading to negative histology. In such cases, serial postoperative measurement of serum hCG should be employed to confirm that the ectopic pregnancy has been successfully treated (Farahani *et al.*, 2017).

Molar pregnancies have been reported in tubal, ovarian, and cervical locations, but overall this is rare (Aytan *et al.*, 2008; Church *et al.*, 2008; Hosseini *et al.*, 2023). However, it has been demonstrated that the extravillous proliferation will appear more florid in TEPs compared to evacuated uterine products of conception, and, therefore, there is a higher risk of overdiagnosis. Only 6% (8/132) of cases referred to a regional referral centre (one of three UK centres) with suspected molar histology over an 18-year period were ultimately confirmed as such (compared to 90% for routine uterine curettage) (Sebire *et al.*, 2005). None of these required any additional treatment.

Although histology is not available after successful expectant or medical management, protocols follow-up patients until normalization of hCG, meaning any persistent gestational trophoblastic disease would be picked up (Sebire *et al.*, 2005).

## Management

The first recorded survival after a ruptured TEP was in 1883, following surgery performed by Robert Lawson Tait (Lurie, 1992). The same surgeon is credited with the first record of delivering an abdominal pregnancy 6 years prior. The first major progress in survival from the condition came in the 1940s, when blood transfusion became available: prior to this, proposed treatment options ranged from the bizarre (starvation) to the outright harmful (intentional exsanguinations of the woman) (Marion and Meeks, 2012).

Advances in ultrasound detection, and access to scans, have enabled smaller ectopic pregnancies to be identified. This has facilitated wider use of conservative management and underlies the importance of avoiding unnecessary intervention, potentially resulting in direct iatrogenic harm or long-term implications for fertility. Early diagnosis in stable patients also enables discussion of the benefits and risks of each management approach, enabling patient-led decision-making. The patient’s willingness and availability for follow-up may impact the appropriateness of expectant or medical approaches. Whether the current pregnancy or future pregnancies are desired may also impact management decisions.

By contrast, in those who present acutely with evidence of significant blood loss, surgical management is imperative. This is more likely in LMIC, where access to skilled ultrasonography is limited and thus timely diagnosis is less feasible. An overview of management options is shown in Table 4.

**Table 4.** Possible management approaches depending on location of ectopic pregnancy.

Diagnosis	Conservative treatment	Surgical treatment
**Tubal**		
Tubal (isthmic, ampullary or fimbrial)	Expectant Methotrexate	Salpingectomy Salpingotomy
Complete interstitial	Expectant Methotrexate	Cornuotomy and repair, with ipsilateral salpingectomy
Partial interstitial		Transcervical suction evacuation
**Other extrauterine**		
Ovarian	*(conservative treatment rarely appropriate)*	Excision/wedge resection of ovary
Abdominal	Expectant Methotrexate	Excision of abdominal pregnancy
**Uterine**		
Partial Caesarean scar or cervical	Expectant Methotrexate	Transcervical suction evacuation ± haemostatic measures Balloon compression Hysteroscopy Laparoscopic/open transabdominal excision Hysterectomy
Complete Caesarean scar or cervical		Laparoscopic/open transabdominal excision Hysterectomy
Partial intramural	Expectant Methotrexate	Transcervical suction evacuation ± haemostatic measures
Complete intramural		Hysterotomy, evacuation and repair
**Within uterine anomaly**		
Non-communicating rudimentary horn	*(conservative treatment rarely appropriate)*	Excision of rudimentary horn
Communicating rudimentary horn	Expectant	Transcervical suction evacuation Excision of rudimentary horn

For conservative management of live ectopic pregnancies, local administration of methotrexate is generally more effective than systemic treatment. Local administration of methotrexate into a pregnancy may be considered if surgical access is limited, and there is safe transvaginal or transabdominal access into a relatively immobile structure. It is also important that the ectopic is contained within myometrium or dense adhesions, such that any resulting bleeding during resolution is likely to be contained, with resulting tamponade. However, local treatment with methotrexate is not appropriate for live isthmic, ampullary, and fimbrial tubal ectopic pregnancies: in these cases, surgery is the preferred treatment option.

Women with a live uterine ectopic pregnancy, particularly Caesarean scar ectopic pregnancy, may elect to continue with the pregnancy having been fully informed about the risks of doing so.

### Management of tubal ecoptic pregnancy

#### Expectant management

As ultrasound resolution and access to scans improve, there will be an increasing number of TEPs that are amenable to expectant management. Expectant management, first proposed in 1955 (Lund, 1955), is now recommended in specific circumstances in both UK and USA guidance: namely, in asymptomatic or mildly symptomatic patients with objective evidence of resolution (small pregnancies with plateauing or declining hCG levels, and excluding live pregnancies) (RCOG, 2016; ACOG, 2018). Overall, 31–40% of TEPs can be expected to resolve spontaneously without needing any intervention, with reassuring evidence regarding safety, provided that robust protocols for follow-up are in place (Elson *et al.*, 2004; Mavrelos *et al.*, 2013; Solangon *et al.*, 2023). Expectant management has also been successfully used in selected interstitial pregnancies (Poon *et al.*, 2014).

The lower the hCG threshold chosen for inclusion, and the smaller the pregnancy appears on ultrasound, the higher the success rate of any protocol. Authors have proposed hCG cut offs of 1000 IU/l (Trio *et al.*, 1995) and 1500 IU/l (Elson *et al.*, 2004), while others have found that a higher threshold of 3000 IU/l (Takashima *et al.*, 2009) is also compatible with high success rates, provided a 20% decline within 48 h is observed. Taking the available evidence into account, UK guidance suggests expectant management should be offered to clinically stable women with ectopic pregnancies <35 mm (without a live embryo) and with HCG <1000 IU/l, and considered at hCG 1000–1500 IU/l (a range in which there is less evidence) (NICE, 2023). The initial change in hCG also determines success, with a plateau or spontaneous decline being a criterion for expectant management in American guidance (ACOG, 2018). A sustained increase, or an hCG climbing above 2000 IU/l, is considered a reason to discontinue expectant management (Kirk *et al.*, 2011; Mavrelos *et al.*, 2013). A higher progesterone level and larger size are also predictors of failure of expectant management (Elson *et al.*, 2004).

Patient selection must also take into account their ability for, and the acceptability of, prolonged follow-up, with an average follow-up of 15 days (range 3–66) (Elson *et al.*, 2004).

Ultrasound resolution occurs in almost two-thirds of patients within 2 weeks of normalization of hCG, and in 95% within 78 days (Dooley *et al.*, 2020). There is also evidence that tubal patency is restored quickly; in one study from Finland, hysterosalpingography confirmed ipsilateral patency in 28/30 patients at an average of 30 days after expectant management (Rantala and Makinen, 1997).

#### Medical management

Medical management aims to promote resolution of an ectopic pregnancy by arresting growth and development. It is therefore only a consideration in stable and minimally symptomatic patients.

The systemic agent most used in ectopic pregnancy is methotrexate, a folate antagonist that acts by inhibiting rapid cell division. It is essential, prior to any consideration of the drug, to rule out any possibility of a potentially viable, correctly sited pregnancy, to which it would be toxic. The UK national guidance suggests that any ectopic visualized as a solid swelling should not be given methotrexate after a single scan unless follow-up hCG levels are consistent with an ectopic pregnancy (RCOG, 2016).

Guidelines originating from the UK, Ireland, Canada, and America offer similar indications and contraindications for methotrexate, requiring the pregnancy to be small, not containing a live embryo, and with relatively low serum hCG levels (<5000 IU/l) (Tsakiridis *et al.*, 2020). A spontaneous drop in hCG levels prior to administration indicates that expectant management is likely to be more appropriate. Protocols suggest a dosage calculated according to body surface area (usually 50 mg/m^2^). hCG levels are then monitored 4 and 7 days after administration. If a drop of <15% in hCG levels is seen, consideration can be given to a second dose. hCG monitoring should continue weekly thereafter until levels are <15 IU/l (RCOG, 2016; ACOG, 2018). Overall, women with TEP with hCG levels between 1000 and 5000 IU/l take a median of 28 days for biochemical resolution, require a second dose in 14% of cases, and have surgical management in 29% of cases (Horne *et al.*, 2023). A higher pre-treatment hCG ratio (2 days apart) has been shown to be associated with increased risk of treatment failure (Kirk *et al.*, 2011). As it is teratogenic, women are advised to avoid further pregnancy for 3 months.

Systemic methotrexate has been compared in a randomized trial to salpingotomy; success rates were shown to be high in both, with no significant difference in patency of the affected Fallopian tube on hysterosalpingography at follow-up (Hajenius *et al.*, 1997). Costs were found to be lower for systemic methotrexate at hCG <1500 IU/l, but higher at >3000 IU/l (and similar between these values): higher costs reflect both productivity loss and the high cost of reintervention (Mol *et al.*, 1999). Another economic evaluation comparing laparoscopy (11/28 having salpingectomy) found that any reduction in indirect costs with methotrexate is lost at hCG >1500 IU/l (Sowter *et al.*, 2001). A population-based study including the cost of subsequent fertility treatment demonstrated that methotrexate was cost-effective compared to surgery, both in terms of direct costs and a reduction in resource allocation to achieve a subsequent correctly-sited pregnancy (Seror *et al.*, 2007).

Other studies have compared methotrexate to expectant management. In contrast to historical observational data, a recent meta-analysis demonstrated no significant benefit for methotrexate compared to expectant management for resolution of TEP, both locally and systemically, and in single-dose or multi-dose regimens (Al Wattar *et al.*, 2024). Given the potential risks associated with systemic methotrexate administration, including liver cirrhosis, renal failure, bone marrow suppression, and pulmonary fibrosis, it seems likely that many clinicians will move away from routinely recommending this treatment over time.

The recently published GEM3 study, in which women were randomized to 7 days of oral gefitinib (an epidermal growth factor receptor agonist) or placebo in addition to intramuscular methotrexate, demonstrated no clinical benefit of gefitinib (Horne *et al.*, 2023). A further randomized study assessing the value of adding mifepristone to methotrexate is suggested, following promising results from small studies (Gazvani *et al.*, 1998; Rozenberg *et al.*, 2003; Oltman *et al.*, 2023).

Although local methotrexate administration directly into the pregnancy has been described for TEPs (Feichtinger and Kemeter, 1987), transvaginal puncture into mobile isthmic and ampullary ectopics is technically difficult with a high risk of complications. Local treatment is easier and safer in cases of interstitial pregnancies. Indeed, prior to the advent of safe operative laparoscopy, local injection of methotrexate was the preferred treatment of live interstitial pregnancies. A series described the successful use of a single, ultrasound-guided, transvaginal injection of 25 mg methotrexate into the GS or chorionic tissue (±0.2–0.4 mEq potassium chloride intracardially to live pregnancies) in 21/23 (91%) cases (Cassik *et al.*, 2005). However, the use of methotrexate for this indication has reduced in recent years and is now limited to small pregnancies, which are located deep within the myometrium and therefore difficult to access surgically.

Other agents that have been investigated for local administration include glucose and prostaglandin; neither of these have demonstrated superiority over expectant management to achieve resolution (Al Wattar *et al.*, 2024).

#### Surgical management

Salpingectomy is the standard surgical treatment, and is the first-line treatment in any patient presenting with haemodynamic instability. Where possible, a laparoscopic approach should be used.

As early as 1922, consideration was given in the literature to whether routine removal of the tube is justified or whether a more conservative approach might offer better fertility prospects (Whitehouse, 1922). The risks of doing so in terms of persistent trophoblast and recurrence were also noted in early studies (Rosenblum *et al.*, 1960; Richards, 1984). The ESEP study was a randomized controlled study comparing salpingostomy and salpingectomy, excluding women with significant contralateral tubal pathology (Mol *et al.*, 2014). The cumulative ongoing pregnancy rate at 3 years was non-significantly higher after salpingotomy (61% versus 56%), with a significantly higher risk of persistent trophoblast (7% versus <1%) and a non-significantly higher risk of repeat ectopic pregnancy (8% versus 5%). Of the women allocated to salpingotomy, 20% required salpingectomy due to persistent bleeding. Based on these findings, salpingotomy was not found to be cost-effective over salpingectomy, with additional costs associated with reintervention (Mol *et al.*, 2015). By contrast, a population-based study in France included a consideration of the additional cost of fertility treatment to achieve a subsequent pregnancy and suggested conservative surgery was cost-effective on this basis (Seror *et al.*, 2007). A further randomized study of radical and conservative surgery performed across France demonstrated similar correctly sited pregnancy rates at 2 years (Fernandez *et al.*, 2013). The UK guidance currently suggests removal of the Fallopian tube as standard in those with a healthy contralateral tube (RCOG, 2016; NICE, 2023). However, the possibility that improvements in surgical techniques since these studies might mitigate trauma during salpingotomy, and be associated with better outcomes, should be considered and ultimately re-explored.

Salpingotomy is broadly recommended if there is contralateral tubal damage, previous abdominal surgery, previous PID, or previous ectopic in order to maximize the possibility of future natural conception, albeit with a higher risk of reintervention or recurrence (RCOG, 2016). Patients may also prefer a conservative approach, worrying about a future ectopic rendering them infertile if a second tube is removed. Conversely, salpingectomy may be preferred if the patient is certain their family is complete. In those desiring future pregnacy, it may be appropriate to consider their access to IVF or involve fertility specialists for a realistic appraisal of its success so that this can be incorporated into decision-making, with a lower threshold for radical surgery in those planning IVF. The appropriateness of conservative surgery also relies on nuanced intra-operative assessment by the surgeon, considering both the extent of pre-existing or intra-operative damage to the tube (including from thermal tissue damage to achieve coagulation) and the difficulty of any repeat operation should it be required for either residual tissue or a future ectopic. Salpingotomy may also require more surgical expertise, which may not be available out of hours.

Following salpingotomy, follow-up with serum hCG testing, until at pre-pregnancy levels, is required, and the willingness and availability for such follow-up must also be incorporated into the decision on surgical approach. Increases in hCG during follow-up suggest residual active tissue, and additional medical or surgical treatment is usually required. Larger ectopic pregnancies and location in the fimbrial or isthmic region have been found to be associated with a higher failure rate of salpingotomy in one study (Kayatas *et al.*, 2014), while another showed no association of any recorded clinical variables and failure (Lund *et al.*, 2002). Adjuvant treatment with methotrexate following salpingotomy may also be of some benefit; a combination was found to offer a better chance of resolution compared to salpingotomy or methotrexate alone across three studies included in a meta-analysis with moderate risk of bias (Al Wattar *et al.*, 2024).

Regarding surgical technique used for salpingectomy, the use of a pre-tied laparoscopic suture has been shown in a randomized study to be associated with a shorter operating time (from a mean of 61 min to 49 min), and may minimize the risk of thermal injury from electrocautery (Lim *et al.*, 2007). Cost may also be lower. A retrospective study of 98 patients comparing salpingectomy and salpingotomy with and without suturing found no significant difference in future correctly sited pregnancy rates over 3 years (Zhang *et al.*, 2022). A novel approach using transvaginal natural orifice transluminal endoscopic surgery (V-NOTES) to perform vaginal salpingectomy has been proposed and has been reported to have equivalent safety and efficacy to a traditional approach, with lower postoperative pain and a better cosmetic outcome (Xu *et al.*, 2014; Lamblin *et al.*, 2021). Approximately 4% patients require conversion to open surgery, often due to adhesions between the uterus and the rectum (Liu *et al.*, 2024).

Interstitial pregnancies may be surgically more challenging, both due to location and later presentation. Cornual resection (also known as wedge resection) has been advocated in the past but is known to disrupt the architecture of the uterus, and is associated with significant rupture risk in a subsequent pregnancy, which was as high as 30% in one series (Liao *et al.*, 2017). Instead, a cornuotomy and removal of ectopic gestation should be advocated; this less-invasive approach is associated with high success and shorter operating time (Choi *et al.*, 2009; Watanabe *et al.*, 2014; Lee *et al.*, 2017; Jin *et al.*, 2020). Localization of the pregnancy using intra-operative ultrasound may occasionally be needed. A salpingectomy should generally be simultaneously performed. A pre-tied laparoscopic suture applied around the cornu may be helpful in applying a temporary tamponade, and vasopressin may also help minimize blood loss and maintain a clear operative field (Elsherbiny *et al.*, 2023).

The UK guidance suggests that Anti-D be given to Rhesus-negative women based on historical evidence of foetal cells in the maternal circulation following ruptured TEP (Katz and Marcus, 1972; RCOG, 2016).

### Management of other extrauterine ectopic pregnancy

Surgical management is recommended for ovarian ectopic pregnancies and can usually be achieved laparoscopically (Fig. 3) (Ko *et al.*, 2012; Solangon *et al.*, 2024). The pregnancy can be excised, or the affected wedge of the ovary can be resected. Blood loss is higher than during surgical management for TEP, with a mean blood loss of 700 ml in one series (Solangon *et al.*, 2024). Following surgical management, serial hCG measurement is indicated to ensure there is no active residual trophoblast.

The management of abdominal ectopic pregnancies will depend on the location, the gestation at which it is diagnosed, symptoms at diagnosis, and the viability of the pregnancy. Medical management does not have a major role, and generally surgical management is required, with a risk of intra-operative haemorrhage and the need for blood transfusion (Poole *et al.*, 2012). Essential to the safe management of abdominal pregnancies is preoperative diagnosis, to enable the presence of senior surgeons (Atrash *et al.*, 1987).

### Management of uterine ectopic pregnancy

Individualized management of uterine ectopic pregnancy is determined by two important factors: (i) whether it is live or failed and (ii) whether it is partial or complete. The former will determine whether spontaneous regression is likely or whether the pregnancy could progress to reach viability, and the latter determines what surgical access can be achieved.

#### Caesarean scar ectopic pregnancy

CSEP is part of a spectrum of abnormal placentation, which may have potential for live birth (Timor-Tritsch *et al.*, 2014). Some women will continue their pregnancy, acknowledging the high risk of hysterectomy at delivery and the simultaneous potential for life-threatening haemorrhage, late miscarriage, or extreme prematurity. In many places, they will be supported to terminate the pregnancy, given the risks to both mother and baby.

A systematic review and meta-analysis of CSEP managed expectantly demonstrated that, out of 17 women with CSEP without cardiac activity, the majority (12/17, 69%) experienced uncomplicated miscarriage, but there was one case of uterine rupture and four cases of complicated miscarriage (Cali *et al.*, 2018). In those with cardiac activity, 12/39 (30%) miscarried, with 4/39 (13%) women experiencing severe vaginal bleeding and 6/39 (15%) requiring hysterectomy in the first or second trimester. In a UK cohort study, nine cases had foetal cardiac activity and were managed expectantly: five progressed to live births, and of the remainder, two resolved, one required surgical evacuation, and one case of a heterotopic pregnancy required a hysterectomy at 17 weeks (Harb *et al.*, 2018). The Society for Maternal–Fetal Medicine has recommended against expectant management of CSEP, except where there is evidence of early pregnancy failure, when serial surveillance may be appropriate (noting the potential for prolonged follow-up) (Society for Maternal–Fetal Medicine *et al.*, 2022).

If treated, management of CSEP will depend on features of the pregnancy itself, as well as the patient’s symptoms and preferences, and local expertise. Management with systemic methotrexate was effective in only 59% in an international registry study and associated with a high risk of complications and is therefore not recommended (Society for Maternal–Fetal Medicine *et al.*, 2022; Kaelin Agten *et al.*, 2024). Intragestational methotrexate can be considered, with a reported success rate of 74%, but resolution can take months (mean resolution time of 88 days was reported in one study) (Timor-Tritsch *et al.*, 2012; Cheung, 2015). Local potassium chloride has been used in the management of heterotopic (one CSEP, one eutopic) pregnancies, with later live birth of the eutopic pregnancy. Balloon catheter compression to treat early CSEP has also been described (Timor-Tritsch *et al.*, 2016).

The mainstay of CSEP management is surgical (Harb *et al.*, 2018). Blood supply from large arcuate and helicine arteries, along with loss of myometrium in the affected area, leading to reduced contractility, results in the potential for massive blood loss (Nijjar *et al.*, 2023). There is no universally agreed surgical strategy, and considerable heterogeneity in the methods is reported in the literature. Comparison of strategies is difficult since circumstances appropriately dictate the surgical method used. Providing the pregnancy is partial, i.e. with extension into the uterine cavity, transcervical evacuation can be performed with suction, under transabdominal or transrectal ultrasound guidance, with excellent success and low complication rates (Jurkovic *et al.*, 2016; Kaelin Agten *et al.*, 2024). Additional haemostatic measures are required in a high proportion of cases, and uterotonics (misoprostol and syntometrine) are commonly used. In a UK cohort study of 56 patients managed with transcervical evacuation, 19 required a Shirodkar suture, and 7 were treated with a Foley catheter to provide tamponade (Harb *et al.*, 2018). Recommendations advise against the use of sharp curettage due to the risk of severing deep blood vessels and a reported 52% reintervention rate (Birch Petersen *et al.*, 2016; Society for Maternal–Fetal Medicine *et al.*, 2022).

Treatment before 9 weeks’ gestation, crown–rump length <23 mm, and lower vascularity on Doppler examination are associated with lower complexity and significantly lower likelihood of blood transfusion (Nijjar *et al.*, 2023). A series of 17 patients with advanced CSEP above 10 weeks’ gestation were managed effectively with transcervical evacuation and insertion of a Shirodkar suture. Uterine artery embolization was available as an adjunct to manage any ongoing bleeding after the Shirodkar suture was tied and was needed in 4/17 patients (Nijjar *et al.*, 2024). All patients avoided hysterectomy.

Hysteroscopic resection has been utilized, with proponents citing high success rates (Di Spiezio Sardo *et al.*, 2023). A systematic review including 10 studies, all of which originated from China and with a mean gestational age of 8 weeks, reported a high success rates, ranging from 65 to 96%, and low complication rates from hysteroscopy (Diakosavvas *et al.*, 2022). However, hysteroscopy would not be feasible in later pregnancies or in those that herniate into the broad ligament. Furthermore, views are often hampered by active bleeding, with a high risk of bladder injury, and distention of the uterine cavity with fluid may counteract attempts to contract it for haemostasis. A recently published large international registry included 460 cases of CSEP, 258 of which were managed surgically (Kaelin Agten *et al.*, 2024). Hysteroscopy was used only in 5/258 (2%) of surgically managed cases. In two of these cases, the procedure was converted to a laparotomy and hysterectomy.

Laparoscopic or open hysterotomy with excision of CSEP has also been described, particularly for those growing towards the bladder or broad ligament, but is not considered mainstream treatment (Verberkt *et al.*, 2023; Kaelin Agten *et al.*, 2024). Laparoscopic treatment requires considerable surgical expertise and would be extremely challenging if brisk bleeding were encountered. It may be appropriate for complete CSEP but is unlikely to have a major role in partial CSEP, where transcervical access is much less invasive. Open surgery may be considered, but with the expectation of a longer recovery time. Transvaginal hysterotomy has also been described in limited case series, but is not a widespread practice (Huanxiao *et al.*, 2015). Hysterectomy is generally considered an emergency adjunct in cases of intractable bleeding, without access to embolization. It may occasionally be considered electively in patients who present late in the second trimester of pregnancy (Society for Maternal–Fetal Medicine *et al.*, 2022).

#### Cervical ectopic pregnancy

Treatment of cervical pregnancy follows the same principles as the treatment of CSEP and includes both medical and surgical management options. Cervical pregnancies are located closer to the external os than CSEP, and therefore they tend to become symptomatic at an earlier gestation and are much less likely to continue for long enough to reach viability (Alalade *et al.*, 2017). The lack of contractility of the cervical stroma following trophoblast or placenta separation results in a significant risk of haemorrhage (Fowler *et al.* 2021). Reported medical techniques include the use of systemic methotrexate or local potassium chloride. Surgical techniques include suction curettage or hysteroscopic resection, which are typically combined with additional measures to secure haemostasis such as balloon tamponade, suture, and uterine artery embolization (Jha and Jafeesha, 2023). A scoping review identified 454 cases in the literature between 2000 and 2018. Success rates ranged from 58% for methotrexate to 87% for a combination of surgical evacuation and uterine artery embolization (Fowler *et al.*, 2021). Overall, 41 (9%) required hysterectomy.

#### Intramural ectopic pregnancy

Surgical management may be performed transcervically for partial intramural pregnancies, using suction evacuation under continuous ultrasound guidance, and was appropriate in 7/18 patients in one series (Nijjar *et al.*, 2023). An abdominal, often laparoscopic, approach is required for complete intramural pregnancies, which may be indicated as an emergency when the presentation is with rupture (Ntafam *et al.*, 2024). Evacuation of the trophoblast, which may require localization by ultrasound, and repair of the myometrium are needed.

Expectant management may also be appropriate in intramural ectopic pregnancies in which the hCG is declining; it was successful in 6/18 patients in the aforementioned series (Nijjar *et al.*, 2023). Biochemical and ultrasound resolution can be expected to take time, with a median time of 71 and 214 days, respectively (Nijjar *et al.*, 2023). Embryocide with potassium chloride or direct administration of methotrexate are other options that can be considered for active non-surgical treatment (Ntafam *et al.*, 2024).

#### Ectopic pregnancy within a uterine anomaly

Women diagnosed with unicornuate uterus with functional non-communicating rudimentary horn are at high risk of rudimentary horn pregnancy. A recent large study of 326 women with unicornuate uteri reported that 17% of the women with a functional horn developed rudimentary horn pregnancy (Tellum *et al.*, 2023). If diagnosed prior to pregnancy, surgical excision of a functional horn can be considered to prevent this potentially serious form of ectopic pregnancy. If the attachment is thick, and removal is likely to incur a myometrial defect, the risk of future uterine rupture must also be taken into account.

Where rupture has occurred, control of bleeding by the most expedient method is required. In less acute situations, laparoscopic excision of the rudimentary horn, including the pregnancy within and the Fallopian tube, can be performed (Shin *et al.*, 2024).

Published experience of management of ectopic pregnancy within Roberts’ uterus is scant. Prior to pregnancy, the anomaly may be associated with symptoms or subfertility and has been successfully treated with hysteroscopic metroplasty, removing the obstructing septum (Gulino *et al.*, 2024). Although there are a couple of case reports showing good pregnancy outcomes following surgical correction of Robert’s uterus, in the absence of any other data, we believe that live pregnancy in untreated Robert’s uterus should be managed in a similar way as rudimentary horn pregnancy (Vural *et al.*, 2011; Khunda *et al.*, 2025).

## Long-term care

### Ongoing Caesarean scar, intramural, abdominal, and rudimentary horn pregnancy

If a decision is made to continue a progressive live ectopic pregnancy, the ongoing care of the pregnancy should be in collaboration with a specialist obstetric team with experience in managing abnormal placentation.

CSEP is usually a precursor to placenta accreta syndrome (Ben Nagi *et al.*, 2005; Timor-Tritsch *et al.*, 2014); in a meta-analysis, 40/52 women with CSEP reached the third trimester, of whom 30 (75%) had abnormally invasive placentas, and 23 (58%) required hysterectomies at the time of delivery (Cali *et al.*, 2018). There are scattered case reports of other types of ectopic pregnancy (intramural, abdominal, and rudimentary horn) reaching viability and indeed only being diagnosed at delivery (Fait *et al.*, 1987; Varma *et al.*, 2003; Shin and Kim, 2005; Petit *et al.*, 2013; Rohilla *et al.*, 2018).

When the diagnosis is made pre-labour, the possibility (and likelihood) of rupture, massive haemorrhage (which may be life-threatening or necessitate hysterectomy), miscarriage, or extreme prematurity must be thoroughly explored with the patient. The timing of delivery will weigh up the risks of prematurity against the risk of catastrophic rupture or haemorrhage. The Society for Maternal–Fetal Medicine recommend planned Caesarean between 34 and 35 + 6 weeks of gestation (Society for Maternal–Fetal Medicine *et al.*, 2022). Patients will be advised against travel and to seek immediate help should they experience any bleeding or abdominal pain. Prolonged inpatient management may be required, with a plan for ready access to blood transfusion, interventional radiology, and the required surgical expertise.

In abdominal pregnancies, the complexity of surgery may depend on the site of placentation (and involved organs), the degree of invasion, and the accessibility of the blood supply (Varma *et al.*, 2003). MRI may be helpful in surgical planning (Lockhat *et al.*, 2006). The placenta may appropriately be left *in situ* after ligation of the umbilical cord if attempted removal would be expected to cause catastrophic bleeding (Martin and McCaul, 1990). Long-term management of a retained placenta may include conservative management or a second-stage surgical retrieval after 3 months when the blood flow has usually ceased (Rahaman *et al.*, 2004). The assembled surgical team should be capable of handling complex vascular, bowel, and bladder surgery. Blood transfusion is commonly required, and consideration should be given to preoperative radiological interventions to minimize blood supply (Rahaman *et al.*, 2004; Worley *et al.*, 2008).

### Residual ectopic and aberrant vascular supply

A TEP is termed ‘residual’ if it remains visible on ultrasound 3 months after biochemical resolution of the pregnancy (Kirk *et al.*, 2020). These appear as solid, hypoechoic, and poorly vascularized lesions. The differential diagnosis, which may be of particular concern in those in whom the pregnancy was not previously diagnosed, is of a tubal tumour. The lack of vascularity provides reassurance, and expectant management is appropriate, with the expectation of complete resolution over time.

Primary tubal choriocarcinoma has very rarely been reported and is prone to early metastasis. A serum hCG of >10 000 IU/l may point to this diagnosis; this may be confirmed after surgery and histological examination, after which the hCG may be seen to continue to rise (Xu *et al.*, 2020). Adjuvant chemotherapy is generally highly effective for these tumours.

Retained placental tissue following surgical or medical treatment of uterine ectopic pregnancies usually appears highly vascular on Doppler examination. In such cases, expectant management is appropriate providing patients are asymptomatic or mildly symptomatic.

### Caesarean scar niche management

Elective repair of Caesarean scar defects may be conducted in an attempt to minimize the risk of repeat CSEP or to treat prolonged bleeding or subfertility. Hysteroscopic, laparoscopic, robotic-assisted, vaginal, and open surgery have been reported (Donnez *et al.*, 2017; Mancuso *et al.*, 2021; Vrijdaghs *et al.*, 2022; Gkegkes *et al.*, 2023). It may also be performed at the same time as laparoscopic or open excision of a CSEP (Verberkt *et al.*, 2023). Although case series of such repairs exist, with variable success rates (radiologically and symptomatically), long-term data from randomized trials is lacking (Tulandi and Cohen, 2016; Verberkt *et al.*, 2023), and the recurrence risk of CSEP is sufficiently low that repair for this indication alone is unlikely to be justifiable in a majority of patients (Ben Nagi *et al.*, 2007).

### Subsequent pregnancy

Generally, counselling after one ectopic pregnancy can be optimistic, albeit with discussion of the risk of subfertility and recurrence: most subsequent pregnancies are normally sited.

#### Future fertility

Following TEP, the aetiology originally at play, as well as the ectopic pregnancy and its treatment, are likely to have an adverse effect on fertility overall. Tubal factor subfertility is most likely, but surgical treatment directly to, or close to, the ovary may also have an impact on ovarian reserve. Important to reassure patients, however, is the concept that the remaining Fallopian tube after salpingectomy can compensate for the loss of the other: one study demonstrated that pregnancies after unilateral salpingectomy are generated from ipsilateral ovulation in 32% cases (Ross *et al.*, 2013).

A large cohort study in Scotland demonstrated that women with ectopic pregnancy were less likely than women after miscarriage to have a second pregnancy (adjusted hazard ratio 0.52 (95% CI: 0.5–0.56)), which could reflect lower fertility, though a lower desire for a further pregnancy, perhaps in order to avoid the physical or emotional trauma of recurrence, may also contribute (Bhattacharya *et al.*, 2012). A French registry study conducted over 16 years demonstrated that 70% of women with a previous ectopic pregnancy went on to have a correctly sited pregnancy, and 57% had a live birth, with a mean follow-up of 24 months (de Bennetot *et al.*, 2012).

Other studies have used smaller cohorts of women known to be actively seeking pregnancy. A 1988 Swedish study, which included 112 women who desired pregnancy and were interviewed 5 years after surgically managed TEP, found that 62 (55%) women achieved a eutopic pregnancy, and 60 (54%) had a live birth (Thorburn *et al.*, 1988). A 1996 French study followed up a cohort of 155 women wanting pregnancy after TEP for a mean of 16 months: 92 (59%) achieved a eutopic pregnancy (Job-Spira *et al.*, 1996). These historical studies may reflect generally more clinically severe TEPs, managed surgically, and it would be a reasonable hypothesis that smaller TEPs, managed expectantly, may be associated with better fertility outcomes.

Small observational studies to date have not demonstrated a significant difference in conception or live birth rates comparing surgical, medical, and expectant management (Strobelt *et al.*, 2000; Helmy *et al.*, 2007; Baggio *et al.*, 2021). A randomized study found no significant difference in cumulative fertility comparing treatment with methotrexate to conservative surgery (Fernandez *et al.*, 2013). As explored above, randomized studies have also not demonstrated a clear difference in future pregnancy rates when comparing salpingectomy to salpingotomy (Fernandez *et al.*, 2013; Mol *et al.*, 2014).

In practice, advice about a woman’s individual chances of natural conception will reflect their age and prior reproductive history and ultrasound or surgical findings of endometriosis, adhesions, or contralateral tubal damage. There should be a low threshold to refer to fertility services when there is an added cause for concern about future fertility.

If further pregnancies are not immediately desired, the options for contraception should be explored. Of note, all contraceptive options reduce the risk of further EP compared to not using contraception, and, as such, any can be recommended (Glasier *et al.*, 2019).

#### Recurrence

Women with TEP have been found to be six times more likely to have a further ectopic in a subsequent pregnancy, compared to women with a miscarriage (Bhattacharya *et al.*, 2012). The risk of recurrence is higher in women with a history of subfertility or pelvic inflammatory disease and in those who are nulliparous (Ory *et al.*, 1993; de Bennetot *et al.*, 2012; Ellaithy *et al.*, 2018; Levin *et al.*, 2019). The recurrence risk remains high even if a subsequent pregnancy is attempted via IVF (Weigert *et al.*, 2009). Conversely, recurrence risk may be lower if the initial ectopic pregnancy was associated with IUCD use (Skjeldestad *et al.*, 1998; Bernoux *et al.*, 2000).

Recurrence rates were non-significantly higher after salpingotomy compared to salpingectomy in a randomized trial (8% versus 5%) (Mol *et al.*, 2014). A comparison of recurrence following non-surgical versus surgical management has not been the subject of randomized trials. Differences from observational studies may reflect differences in baseline characteristics, with surgical management generally being associated with larger and more vigorous ectopics. Reassuringly, there is no evidence currently that medical or expectant management for a first ectopic is associated with a higher risk of recurrence than surgical management, in spite of the theoretical concern that partial Fallopian tube blockage or dysfunction may persist (Mackenzie *et al.*, 2023).

Recurrence rates after expectant management are significantly lower if conception takes place soon after a loss (Dooley *et al.*, 2025). Although this may reflect higher fertility as a confounder, it is reassuring that purposeful delay to conception to allow time for physical resolution may not be necessary.

After ovarian ectopic, studies have shown 13/28 (Koo *et al.*, 2011), 15/21 (Wong *et al.*, 2021), and 7/20 (Solangon *et al.*, 2024) eutopic pregnancies at follow-up, with 3/28 in the first study having a TEP (but none reported in the others).

A systematic review of reproductive outcomes following CSEP found that 76% were able to become pregnant again (Wu *et al.*, 2021). The recurrence rates varied widely between different studies, which may reflect the lack of standardized diagnostic criteria. A systematic review reported an average recurrence rate of 18% (Morlando *et al.*, 2023). In 18 cases of intramural ectopic, 4 went on to have a eutopic pregnancy during follow-up, and 1 had a CSEP (Nijjar *et al.*, 2023).

#### Management of recurrences

A second salpingectomy to manage a contralateral TEP recurrence will cause absolute infertility, and a patient who will be reliant on IVF for any future pregnancies, with significant financial and psychological implications. Decisions to attempt to conserve the affected tube may depend on their attitudes towards, access to, and likely success from, IVF, as well as their tolerance for recurrence.

Recurrences in the ipsilateral tube may prompt surgical intervention even in spontaneously resolving cases. One small study demonstrated that when repeat ipsilateral ectopics are managed expectantly, there is a higher risk of repeat ectopic (60%) compared to medical (29%) and surgical management (24%) (Karavani *et al.*, 2021). However, it is important to recognize that salpingectomy does not preclude ipsilateral recurrence, with one series demonstrating that 3/37 recurrences after salpingectomy were in the ipsilateral tube (two in the interstitial portion and one in the tubal stump) (Dooley *et al.*, 2025).

#### Future pregnancy outcome

One large study from Scotland demonstrated no higher risk of pre-eclampsia, pre-term delivery, or emergency Caesarean section in those with a previous ectopic than in those with no previous pregnancies or those with previous miscarriage or termination (Bhattacharya *et al.*, 2012). Another large registry study from Canada reported a higher risk of low birth weight, pre-term delivery, and Caesarean section in those pregnant after an ectopic first pregnancy, managed surgically, compared to those with a eutopic first pregnancy (Chouinard *et al.*, 2019). The authors considered if this may be related to the higher incidence of subfertility, endometriosis, and pelvic surgery in those with ectopic pregnancies. Older women with past ectopic appeared at particularly high risk of placental disorders (Chouinard *et al.*, 2019).

### Psychological outcomes

One study of 150 women with TEP from Iran demonstrated that 76% of women after completion of treatment for ectopic pregnancy met criteria for mental distress and 35% for depression (Hasani *et al.*, 2021). All women in the study reported social dysfunction, and mental distress was significantly associated with low self-esteem. A UK study involving 116 women with TEP demonstrated that 21% of respondents met criteria for post-traumatic stress (PTS), 23% for moderate/severe anxiety, and 11% for moderate/severe depression at 9 months (Farren *et al.*, 2020). Partners were also shown to be affected, albeit in a small group of 17 partners after ectopic pregnancy, with corresponding figures for PTS of 8% and anxiety of 15%, with none meeting criteria for depression (Farren *et al.*, 2021). In a study from Nigeria including 202 women with all types of pregnancy loss, including ectopics, 17% had moderate/severe symptoms of depression (Obi *et al.*, 2009).

Diagnostic uncertainty, including the diagnosis of PUL, has been associated with higher anxiety levels than a certain diagnosis 48–72 h after a scan (Richardson *et al.*, 2017). The potential impact of clinical severity (for example, the need for emergency surgery for patients in extremis) has not been thoroughly investigated in a subgroup of women experiencing ectopic, although research involving all early pregnancy losses indicates psychological reactions may be hard to predict (Farren *et al.*, 2022).

One randomized study compared systemic methotrexate to laparoscopic salpingectomy with respect to health-related quality of life (HRQoL) over 16 weeks; those treated with methotrexate were more depressed, with worse overall quality of life than those treated surgically (Nieuwkerk *et al.*, 1998). A more recent randomized trial found no difference in HRQoL comparing treatment with systemic methotrexate to expectant management in women with ectopic pregnancy or PUL (van Mello *et al.*, 2015).

Although little is known about the impact of CSEP specifically, there is extensive research that demonstrates the significant psychological sequelae associated with termination (Nordal Broen *et al.*, 2005). Thus, an active decision to end the pregnancy, which may be wanted and/or long-awaited, may have added implications. Clinical uncertainty and complexity, with threats of risk to future reproduction or even life, coupled with the requirement to travel for expert management, may also be expected to exacerbate trauma. Other uterine and extrauterine pregnancies may be affected similarly by the potential for live birth or the need for higher-risk, expert management.

Overall, there is evidence of a scale of psychological trauma in response to a common life event that is systemically overlooked. These events occur at a time of peak societal contribution, at work and at home, and such sequelae may have devastating long-term consequences not only for the patient but also for those around them (Lasker and Toedter, 2003). It is likely that some people will abandon future attempts to conceive due to fear of recurrence (Spillane *et al.*, 2018). For others, future pregnancies may be affected by psychological illness, as they are known to be in other contexts, with research linking anxiety and PTS disorder to preterm labour and growth restriction (Mulder *et al.*, 2002; Orr *et al.*, 2007; Rogal *et al.*, 2007; Grote *et al.*, 2010; Yonkers *et al.*, 2014). Bonding with existing or future children may also be impacted; studies have linked maternal depression to poorer long-term socioemotional and cognitive development of children (Bernard-Bonnin, 2004; Bicking Kinsey *et al.*, 2015). Implications may be more devastating still; a study from Israel demonstrated that, out of 160 patients treated with salpingectomy for ectopic pregnancy, 6 had attempted suicide (1 successfully) in the first year after the operation (Farhi *et al.*, 1994). The authors describe the causative combination of ‘an insult to self-image due to failure of the pregnancy, together with the trauma of surgery and the threat to future reproduction’.

Optimal treatment to prevent or treat psychological sequelae is unclear. Historically, a Cochrane review concluded that there was insufficient evidence that psychological support and counselling are effective after miscarriage, but there was a suggestion in all studies of a positive effect, and the need for further research was identified (Murphy *et al.*, 2012). Research specific to ectopic pregnancy is scarce. In one study from China, a ‘comprehensive nursing plan’ involving heath education and physical and emotional support resulted in lower anxiety and depression levels (Zhong *et al.*, 2021). In Iran, a randomized trial with an intervention of four structured counselling sessions demonstrated significant improvement in mental health and self-esteem after 2 weeks (Hasani *et al.*, 2021).

## Scope for future research

Establishing rapid and accurate diagnosis of various types of ectopic pregnancy remains a major challenge, particularly across low-resource settings with limited availability of advanced sonography (Mooij *et al.*, 2018). Several diagnostic models have been proposed to help refine the diagnosis and management of suspected ectopic pregnancies, although widespread evidence of the effectiveness of these models for diagnosis remains limited.

Several of the available medical treatments are focused to avoid the need for salpingectomy and preservation of future fertility (Al Wattar *et al.*, 2024). However, to date, most of the available randomized trials evaluated head-to-head comparisons of various medical treatments without direct comparison with the standard treatment (salpingectomy) or the least-invasive treatment (expectant management). Future trials should focus on evaluating the superiority of emerging treatments for two primary outcomes: (i) full resolution of the ectopic pregnancy and (ii) the risk of needing emergency surgery or additional interventions.

Other key outcomes, such as future fertility and risk of recurrence, are difficult to capture in the context of randomized trials (Baggio *et al.*, 2021). To date, the reporting of these important outcomes has been limited by the lack of long-term, prospective follow-up of randomized cohorts (Hao *et al.*, 2023). As better and more standardized health reporting systems emerge, especially since the COVID-19 pandemic, there is a need to invest in setting up a conducive research infrastructure to capture long-term health outcomes of women with ectopic pregnancy.

Variations in outcome reporting are a common problem hindering effective evidence synthesis, particularly when evaluating long-term outcomes after ectopic pregnancy (Chong *et al.*, 2023). The use of core outcome sets, focused on outcomes prioritized by women, can help to reduce research wastage and optimize impact (Khan, 2014). Specifically, comparing complex interventions with prolonged follow-up often have under-reported the impact on quality of life and treatment satisfaction. Investing in developing and validating condition-specific quality-of-life assessment tools will optimize such evaluations.

Research on appropriate support, counselling, or other interventions to prevent, reduce, or treat psychological sequelae, is needed. Given the frequency of psychiatric morbidity identified, and its potential implications for functioning and across generations, identifying an effective treatment would be likely to have far-reaching impact.

Finally, understanding the differential prevalence in ectopic pregnancy and its outcomes for women of Black and minority ethnic groups is a research priority (Stulberg *et al.*, 2016). The most recent MBRRACE report from the UK and Ireland demonstrated that Black women have three times the risk of death in pregnancy as white women, and addressing this inequality was identified as an important focus (MBRRACE-UK *et al.*, 2024a).

## Conclusions

Ectopic pregnancy is often a seismic event in a woman’s reproductive journey. Aside from generally representing the loss of a pregnancy, it may pose a serious physical threat to the woman and may occasionally be lethal.

Competent, timely ultrasound assessment is the cornerstone of safe care in early pregnancy. A false-positive diagnosis of ectopic can cause harm by instigating unnecessary interventions, which could result in iatrogenic injury to the woman. Uterine instrumentation during surgery, or the administration of methotrexate, may also result in the loss of a normal pregnancy. A basic principle should be that no intervention should be initiated in a stable woman until diagnostic certainty is achieved, with recourse to regional experts if there is difficulty.

Increasingly, through earlier diagnosis and established principles for case-selection and monitoring, expectant management is appropriate. Where surgical intervention is required, minimally invasive techniques should be used, with transcervical evacuation for partial uterine ectopics being the most logical approach. We propose that systemic medical management with methotrexate should no longer be considered first-line management. Other aspects of care include managing the psychological implications of ectopic pregnancy and ensuring timely access to fertility treatment, if required. Efforts going forward should focus on optimizing diagnosis and treatment selection in an effort to minimize both natural and iatrogenic harm.

## Data Availability

Not applicable. There are no new data associated with this article.
